# Quasi-alternating copolymerization of oxiranes driven by a benign acetate-based catalyst

**DOI:** 10.1038/s42004-023-01031-z

**Published:** 2023-10-28

**Authors:** Charlotte Fornaciari, Vincent Lemaur, Dario Pasini, Olivier Coulembier

**Affiliations:** 1 Laboratory of Polymeric and Composite Materials (LPCM), Center of Innovation and Research in Materials and Polymers (CIRMAP), University of Mons, Place du Parc, 20, Mons, 7000 Belgium; 2 Department of Chemistry, University of Pavia, Viale Taramelli, 10, Pavia, 27100 Italy; 3 Laboratory for Chemistry of Novel Materials, Materials Research Institute, University of Mons, Place du Parc, 20, 7000 Mons, Belgium

**Keywords:** Materials science, Green chemistry, Polymer synthesis, Polymers

## Abstract

Alternating copolymers are distinctly unique in comparison with other copolymers. Herein, an in-depth investigation of the oxyanionic ring-opening copolymerization of propylene oxide (PO) and allyl glycidyl ether (AGE) from benzyl alcohol (BnOH) activated with potassium acetate (KOAc) complexed by 18-crown-6 ether (18C6) is described. We demonstrate that the 18C6/KOAc complex is an efficient and benign catalytic system to promote copolymerization of both oxirane monomers, leading to well-defined polyethers with varied comonomer content and low dispersity values (*Ɖ*_*M*_ < 1.20). Kinetic analysis confirmed the controlled nature of the (co)polymerization process, and the determination of reactivity ratios revealed a quasi-alternating copolymerization profile, according to the Fineman-Ross method. The comparison between the quasi-alternating-type PO/AGE copolymerization and block or gradient copolymerization revealed significant differences, to confirm the different sequence incorporation in the different topological copolymers. These results highlight the great potential of 18C6/KOAc-mediated copolymerization process for the controlled sythesis of a series of copolymer topologies.

## all

To meet the rising demand for sustainable and simple polymer syntheses, organocatalytic polymerizations are a powerful tool. Here, the authors report the quasi-alternating polymerization of oxirane monomers at room temperature and in solvent-free conditions catalyzed by potassium acetate complexed by 18-crown-6 ether, leading to well-defined polyethers with varied comonomer content and low dispersity values.

## Introduction

The development of controlled (co)polymerization techniques for epoxides has significantly expanded the scope of polymer science and engineering, offering new opportunities for the synthesis of advanced polymeric materials^1,2^. The ability to precisely control the monomer distribution along the copolymer chain is essential to tailor the physical and mechanical properties, unlocking applications which are not accessible with the homopolymers or random copolymers. Propylene oxide (PO) is one of the most important epoxide monomers, holding a dominant position as starting material processed in the polymer industry on a megaton scale^3^. The oxyanionic ring-opening polymerization (ROP) using alkali metal alkoxide initiators represents the method of choice for preparing poly(propylene oxide) (PPO) and a large fraction of polyethers, including its copolymers^4–6^. In these systems, especially at high temperatures, the strong basicity of the initiating/propagating alkoxides gives rise to irreversible chain transfer processes through the proton abstraction from the methyl group of PO, leading to the formation of an allyl alkoxide, which in turn may acts as an initiator of a new chain, impairing polymerization efficiency and molar mass of the obtained polyethers^4,7–10^. Several polymerization strategies using anionic or coordination catalysts, such as double metal cyanide (DMC) complexes^2,11–13^, tri-isobutylaluminum (i-Bu_3_Al)^14–17^, organic Lewis pair comprising of phosphazene bases and triethylborane (Et_3_B)^18^, successfully afforded well-defined PPO via controlled/living polymerization procedures. Although effective, some of these catalytic systems often required expensive and unstable reagents, complicated handling techniques, and strict temperature control. To meet the rising demand for sustainable and simple ROP systems, the organocatalytic polymerization approach has emerged as a powerful tool for polyether synthesis^19–23^. In this context, alkali metal carboxylate catalysts, eventually complexed by crown ethers^24,25^, have played a crucial role in the development of various (co)polymers due to their ability to be used in bulk, of their low cost and low toxicity. Our group has recently demonstrated the potential of an equimolar mixture of potassium acetate (KOAc) and 18-crown-6 ether (18C6) as a robust and benign catalytic system for preparing PPO in a controlled fashion at room temperature and in solvent-free conditions^26^. In the presence of 18C6/KOAc complex, hydrogen-bonded alcohols act as soft nucleophiles promoting the PO polymerization, while drastically limiting the occurrence of parasitic hydrogen abstraction^27–31^.

The copolymerization of PO with other epoxide monomers, in order to increase chemical functionality within the PPO backbone, remains at the forefront in this field^12,13^. The copolymerization of ethylene oxide (EO) and PO has been extensively studied due to the huge potential of P(EO-*co*-PO) copolymers^12,32–35^. Recent studies, using NMR spectroscopy for monitoring the comonomer consumption during the whole polymerization and for determining the reactivity ratios, have highlighted distinctive differences of the copolymerization behavior of the two monomers depending on copolymerization technique^12^. In the conventional oxyanionic ROP, the copolymerization of EO with PO or glycidyl ethers usually leads to perfectly random copolymers, as it has been established in several kinetics studies^36–39^. Interestingly, the few cases of copolymerizations based on monomer-activated anionic ROP resulted in pronounced gradient structures, as in the case of alkylene oxides and glycidyl ethers, due to the lower reactivity of the former when compared to the latter class^34,40,41^.

The microstructure of the resulting copolymers is reflected in the properties and in the field of applications. For instance, random P(EO-*co*-PO) are used as water-soluble lubricants, preventing foaming due their higher surface tension compared to PEO-*b*-PPO block copolymers^42,43^, while PEO-*b*-PPO-*b*-PEO triblock copolymers are non-ionic surfactants and are used as pharmaceutical ingredients, agricultural products or food additives^44^. Whereas the possibility of gradient copolymerization for P(EO-*co*-PO) has been demonstrated^12^, epoxide-based alternating copolymers have not yet been reported to our knowledge and thus they represent a major challenge. Furthermore, to date, the copolymerization of PO with allyl glycidyl ether (AGE), including the determination of reactivity ratios, is a largely unexplored area.

In this contribution, we report that the quasi-alternating polymerization between oxirane monomers, namely PO and AGE, is indeed possible using the 18C6/KOAc-mediated oxyanionic ROP system at room temperature and in solvent-free conditions. The quasi-alternating copolymerization mechanism is supported by the reactivity ratios of comonomers using the Fineman−Ross method^45^, determined from the monomer consumption during the copolymerization, and by the characterization by combined analyses, including ^1^H and ^13^C nuclear magnetic resonance (NMR) spectroscopy, matrix-assisted laser desorption/ionization time-of-flight mass spectrometry (MALDI-ToF MS), and size exclusion chromatography (SEC) measurements. Using controlled sequential additions of monomer or comonomer feeds at different reaction periods, we report the synthesis of diblock and gradient copolymers of the PO and AGE couple of oxirane monomers, expanding the scope of the oxyanionic ROP system. NMR spectroscopies and MALDI mass spectrometry were used to confirm the differences in the sequence incorporation of monomers between the different topologies (quasi-alternating, diblock, and gradient) of the PO/AGE copolymers. We anticipate that the developed quasi-alternating P(PO-*co*-AGE) copolymer, as well as the block and gradient congeners, will provide a special polyether platforms offering broad scientific appeal encompassing the fields of synthetic and physical polymer chemistry and post-functionalization, biomaterials and medicine, and beyond.

## Results and discussion

### Homopolymerizations of propylene oxide and allyl glycidyl ether

**Fig. 1:**
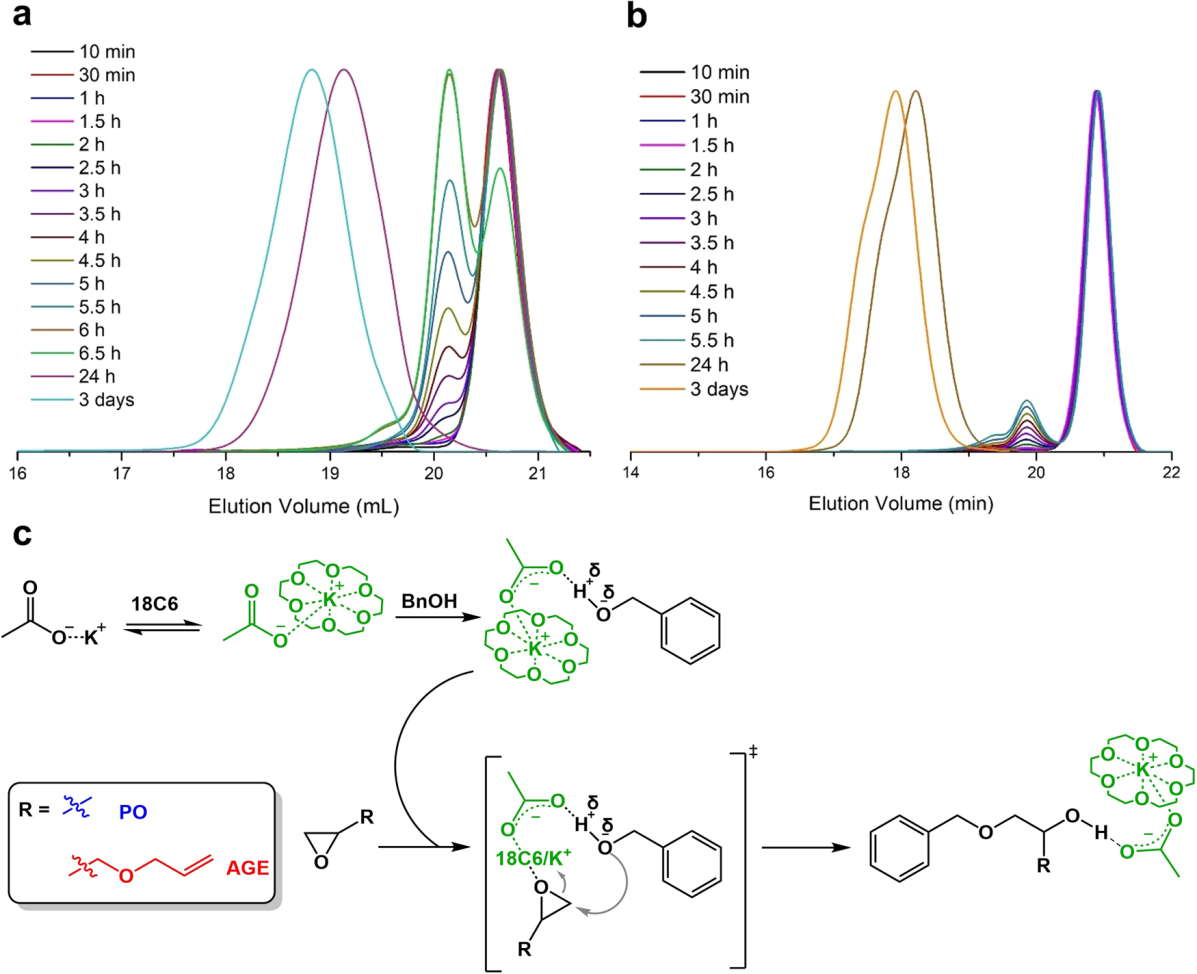
18C6/KOAc-mediated oxyanionic polymerization of oxiranes from benzyl alcohol. Evolution of SEC traces in the homopolymerization of PO (**a**) and AGE (**b**) from BnOH. Conditions: [M]_0_/[BnOH]_0_/[18C6/KOAc]_0_ = 25/1/0.5. **c** Proposed S_N_2 activation mode when PO or AGE are reacted with 18C6/KOAc and BnOH.

Before copolymerizations were undertaken, we first investigated the homopolymerizations of PO and AGE in bulk at room temperature from a benzyl alcohol (BnOH) initiator activated by 18C6/KOAc complex ([BnOH]_0_/[18C6/KOAc]_0_ = 2). Our group previously reported that 15 days were required to homopolymerize PO at 21 °C to get a degree of polymerization (DP = [PO]_0_/[OH]_0_) of 130^26^. Therefore, to reduce the reaction time, low molecular weight PPO and poly(allyl glycidyl ether) (PAGE) were prepared by targeting a degree of polymerization (DP = [M]_0_/[BnOH]_0_, with M, the monomer employed, i.e., PO or AGE) of 25. The consumption of monomers was monitored with time by ^1^H NMR spectroscopy, following the gradual decrease of epoxide methine signals. After 3 h of reaction, NMR analyses revealed that ≈12% of PO and ≈8% of AGE had been converted and a significant amount of unreacted BnOH (≈80%) was still present in both polymerizations (Supplementary Fig. 1). These results strongly suggest the occurrence of a long inhibition period before the actual chain growth can be observed. The SEC measurements provide additional details of the initial events in the polymerization process. During both polymerizations, an intermediate appears to form, detected at around 166 g.mol^−1^ after 2 h and present at least up to 6 h for PO (Fig. 1a) and at 225 g.mol^−1^ for AGE (Fig. 1b). SEC analysis of a mixture of 18C6/KOAc did not match the peak observed for the just mentioned intermediate. A plausible explanation of this phenomena could derive from the formation of a population of one-to-one adduct between BnOH and monomer that persists for a long time as dormant species. To ascertain the identity of the intermediate species, the reaction of PO and BnOH in bulk at room temperature targeting a DP of 2 was studied by a combination of SEC and ^1^H NMR analyses. The product obtained after 2 h shows a molar mass that coincides with that of the species detected in SEC chromatograms during the PO homopolymerization (Supplementary Fig. 2), and this observation was confirmed by NMR analysis (Supplementary Fig. 3). The inhibition period could probably be ascribed to a slow reaction between 18C6 and KOAc, to form the dissociated species AcO^-^ and K^+^/18C6, leading a potassium acetate activator “strongly attached” to Bn-O-CH_2_-CH(R)-OH formed following the first addition (Fig. 1c). Thus, this catalyst/alcohol complex consumes part of the BnOH initiator before to really initiate propagation. Polymerizations proceeded in a near quantitative monomer conversion in 3 days to give a PPO and PAGE with, despite the inhibition step, a monomodal molecular weight distribution and narrow dispersity values (*Ð*_*M*_ = *M*_w_/*M*_n_) of 1.16 for PO (Fig. 1a), and of 1.19 for AGE (Fig. 1b), as determined by SEC. No detrimental effect of proton abstraction has been observed in ^1^H and ^13^C NMR analyses of both final polymers (Supplementary Figs. 4–7).

Interestingly, ^13^C NMR spectrum of PAGE, which consisted predominantly of head-to-tail linkages, was analyzed in terms of triads on the methine carbon on the AGE repeat units, being sensitive to the specific regio- and stereo-configuration of the polymer^46,47^. The deconvolution per the triad signals associated with specific tacticity of the allyl substituents along the backbone revealed that the 18C6/KOAc-mediated ROP produced regio-regular and atactic PAGE with a statistical distribution of 22% isotactic, 25% syndiotactic, and 53% heterotactic triads (Supplementary Fig. 7).

**Fig. 2:**
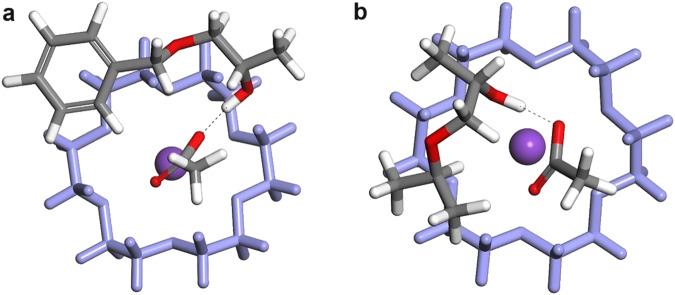
Effect of the initiator structure on the propagating alcohol activation through complexation with the acetate-based catalyst. Representation of the most stable assemblies containing the 18C6/KOAc complex and either a Bn-O-CH_2_-CH(CH_3_)-OH (**a**) and a iPr-O-CH_2_-CH(R)-OH (**b**). The 18C6 crown ether is displayed in purple for clarity.

To determine whether the nature of the initiator molecule impacts the initiation step, we examined the homopolymerizations using isopropyl alcohol (iPrOH) as initiator, while maintaining all other experimental conditions unchanged (conditions: [M]_0_/[iPrOH]_0_/[18C6/KOAc]_0_ = 25/1/0.5). The kinetic studies conducted via ^1^H NMR show a slow inhibition process comparable to the BnOH-initiated polymerization. Interestingly, SEC data suggest that the nature of alcohol influences the polymerization outcome. When BnOH is used as initiator, the one-to-one adduct signal observed in SEC chromatograms remains constant for both monomers, leading to uniform growth chains. In contrast, in iPrOH-initiated polymerization, low molecular weight oligomers begin to form at an early stage of polymerization, without formation or prolonged persistence of an iPr-O-CH_2_-CH(R)-OH adduct, yielding broader molecular weight distributions (Supplementary Fig. 8). Probably the iPrO-CH_2_-CH(R)-OH intermediate species is less stable than the intermediate formed between Bn-O-CH_2_-CH(R)-OH and KOAc and thus more prone to induce monomer propagation. To get more insight on that inhibition period, the relative position between the 18C6/KOAc activating complex and both Bn-O-CH_2_-CH(CH_3_)-OH and iPr-O-CH_2_-CH(CH_3_)-OH one-to-one adducts was evaluated by molecular mechanics and molecular dynamics simulations (Fig. 2) using the COMPASSIII force field as implemented in the BIOVIA Materials Studio 2022 package^48^. It can be inferred that in comparison to the benzyl end-capped adduct (Fig. 2a) the less bulkier isopropyl extremity allows a better nesting of the iPr-O-CH_2_-CH(CH_3_)-OH with the activating complex allowing the hydroxyl extremity to be closer to the activation center (Fig. 2b). The hydrogen-bonding distance is longer for Bn-O-CH_2_-CH(CH_3_)-OH than for the iPr-O-CH_2_-CH(CH_3_)-OH (1.449 Å vs. 1.435 Å) which translates into smaller H-bond interactions for the former (29.3 kJ/mol *versus* 37.7 kJ/mol for Bn-O-CH_2_-CH(CH_3_)-OH and iPr-O-CH_2_-CH(CH_3_)-OH, respectively). Bn-O-CH_2_-CH(CH_3_)-OH adduct appears then as a less activated structure.

Initial studies confirmed the importance of this adduct interaction^26^, since this mechanism occurs entirely through hydrogen bonding between the propagating alcohol end-group and the acetate base. Despite the slow initial 18C6/KOAc/BnOH supramolecular organization by monomer molecules, the intermediate species formed plays a key role in increasing polymerization control and while both homopolymerizations are characterized by a long inhibition period, regardless of the nature of the initiator used, the polymerization kinetics exhibit similar profiles.

### Copolymerizations of PO and AGE

**Fig. 3:**
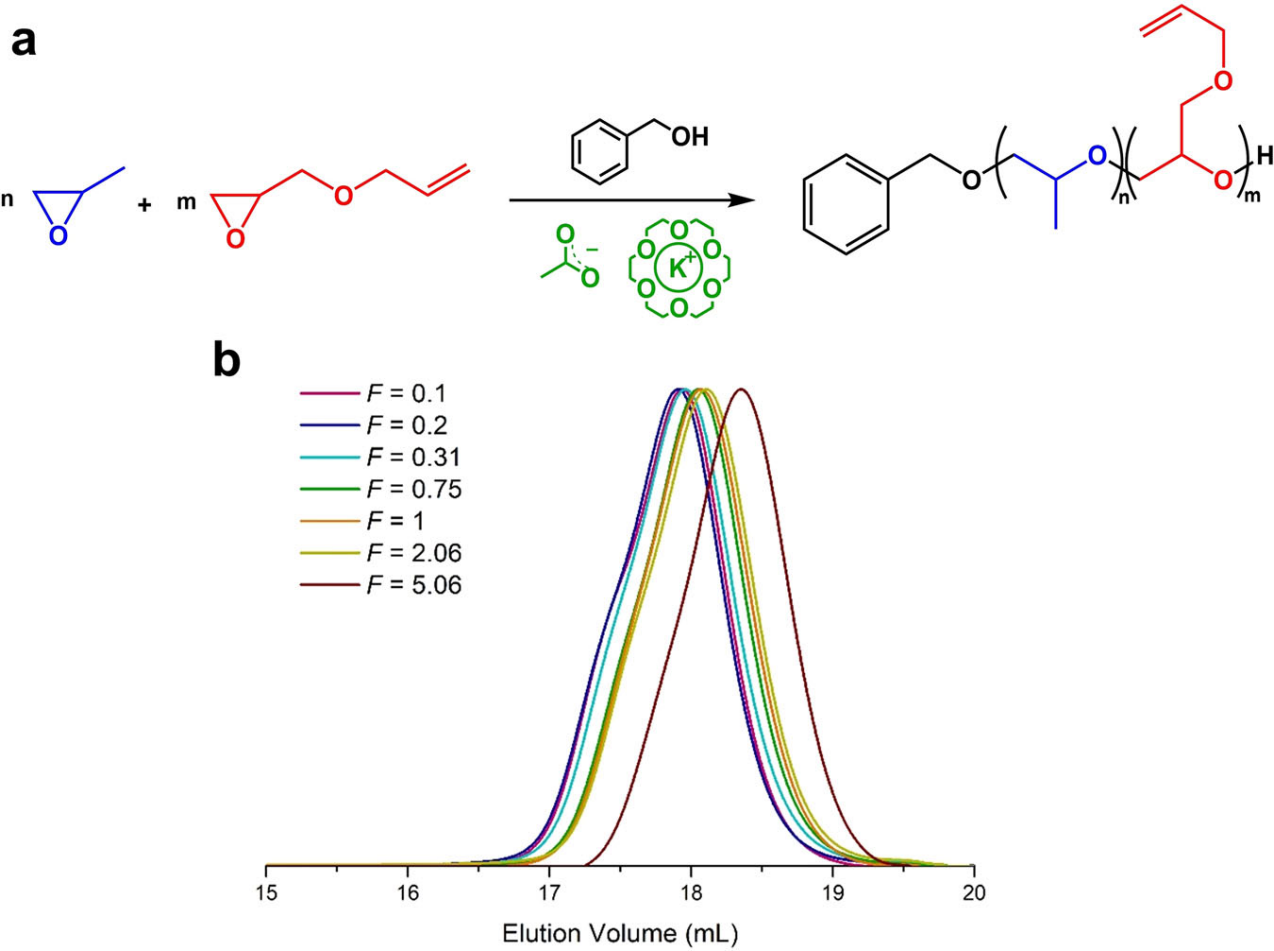
18C6/KOAc-mediated copolymerization reactions of PO and AGE. **a** Scheme of PO and AGE copolymerization from a BnOH activated by 18C6/KOAc complex. **b** SEC traces of the fully converted crude P(PO-*co*-AGE) copolymers synthesized at different molar ratios of comonomers.

Having gained a better understanding of the homopolymerization kinetics using a soft nucleophilic catalysis, we explored the simultaneous polymerization of PO and AGE using BnOH with the following molar mass ratios: [PO + AGE]_0_/[BnOH]_0_/[18C6/KOAc]_0_ = 25/1/0.5 (Fig. 3a). Copolymerizations were carried out in bulk at room temperature until complete consumption of monomers within 7 days. A series of poly[(propylene oxide)-*co*-(allyl glycidyl ether)] (P(PO-*co*-AGE)) copolymers were prepared by varying the initial monomer compositions (*F* = [PO]_0_/[AGE]_0_) (Fig. 3, Table 1 and Supplementary Table 1). SEC traces of the resultant copolymers revealed monomodal molecular weight distributions without a noticeable change in dispersity values (*Ɖ*_*M*_ = 1.16–1.20), attesting an excellent control. Because AGE monomer exhibits higher molecular weight than PO, the number average molecular weight (*M*_*n*_) of the copolymers increases with increasing amount of AGE incorporated, as evidenced by SEC chromatograms (Fig. 3b and Table 1).

**Table 1:** Molecular characterizations of P(PO-*co*-AGE) copolymers obtained at different molar ratios of comonomers.

Entry	*F* = [PO]_0_/[AGE]_0_^a^	${\overline{{{{{{\boldsymbol{DP}}}}}}}}_{{{{{{\boldsymbol{PO}}}}}}}$^b^	${\overline{{{{{{\boldsymbol{DP}}}}}}}}_{{{{{{\boldsymbol{AGE}}}}}}}$^b^	*M*_n_ (g.mol^−1^)^c^	*Ɖ*_*M*_^c^
1	0.1	2	22	1850	1.19
2	0.2	4	20	1860	1.20
3	0.31	6	19	1712	1.20
4	0.75	10	14	1566	1.18
5	1	12	12	1510	1.19
6	2.06	16	8	1467	1.20
7	5.06	19	4	1111	1.16

^a^F = molar fraction of comonomers in the feed.

^b^The degree of polymerization ($\overline{{DP}}$) was confirmed by ^1^H NMR spectroscopy using the ratio between the integration of the methylene protons of the α-phenyloxy copolymers end-group and the allyl or methyl protons of the two different incorporated monomers.

^c^were determined by SEC in THF at 30 °C relative to polystyrene (PS) standards.

**Fig. 4:**
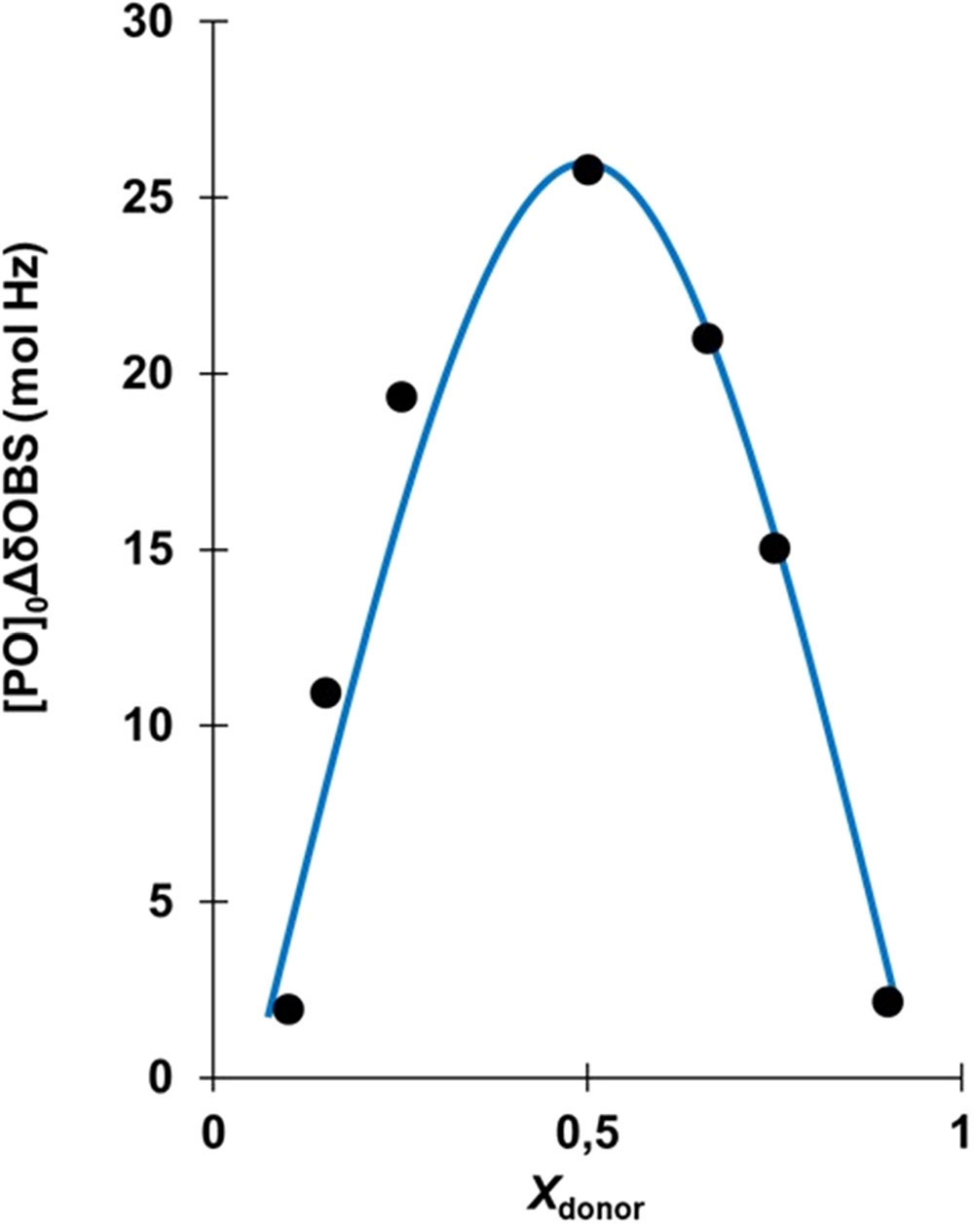
Job’s plot of the NMR chemical shift changes. *X*_donor_ = mole fraction of AGE molecules in PO-AGE mixtures. All analyses were conducted using total mixture concentrations of [PO + AGE] = 8.6 M in THF-*d*_8_ and varying the molar ratio of the two monomers [PO]_0_/[AGE]_0_ = 0.1, 0.3, 0.5, 1, 3, 6, and 9.

In order to ascertain the possibility of specific interaction between PO and AGE, ^1^H NMR analyses using the Job method was carried out^49^, by examining several PO-AGE mixtures of varying compositions using THF-*d*_8_ as solvent, since it dissolves both monomers, it is inert and does not complex with either monomer (Supplementary Fig. 9). From the change in ∆δ of the methine proton of PO in the ^1^H NMR spectra a complex was clearly observed with a 1:1 stoichiometry between PO and AGE (Fig. 4). To some extent the formation of a binary complex between both PO and AGE already supports the acceptable rationale for the tendency of those comonomers to cross-propagate during their copolymerization^50^.

To support our hypothesis, the ability of both PO and AGE monomers to form a binary complex has also been confirmed at the DFT level by investigating the variation in interaction energies among representative dimers, from homo-dimers to hetero-dimers. For each system, we performed quenched dynamics using the COMPASSIII force field to determine their preferential orientations. Subsequently, we estimated the interaction energies of pure PO and AGE homo-dimers, i.e., PO/PO and AGE/AGE, as well as PO/AGE hetero-dimers, based on the lowest energy assembled structures and isolated compounds (all the technical details of the simulations are described in the SI). Our simulations predict an ascending trend in interaction energies, progressing from PO/PO dimers (−3.30 kcal/mol) to PO/AGE dimers (−4.34 kcal/mol), and finally to AGE/AGE dimers (−5.49 kcal/mol). This suggests that PO/AGE systems are more likely to occur than PO/PO homo-dimers.

^1^H NMR kinetic studies of the copolymerization were then performed to gain a deeper understanding of the evolution of the copolymer composition. Taking into account the occurrence of the inhibition period, the kinetic measurements were started after 30 min for *F* < 1, and after 60 min for *F* > 1. By monitoring the methine protons of PO (δ = 2.97–3.01 ppm, m) of AGE (δ = 3.14–3.18 ppm, m), and the characteristic polymer signal of the P(PO-*co*-AGE) backbone (δ = 3.48–3.63 ppm), the respective conversion rates were determined (Supplementary Fig. 10).

**Fig. 5:**
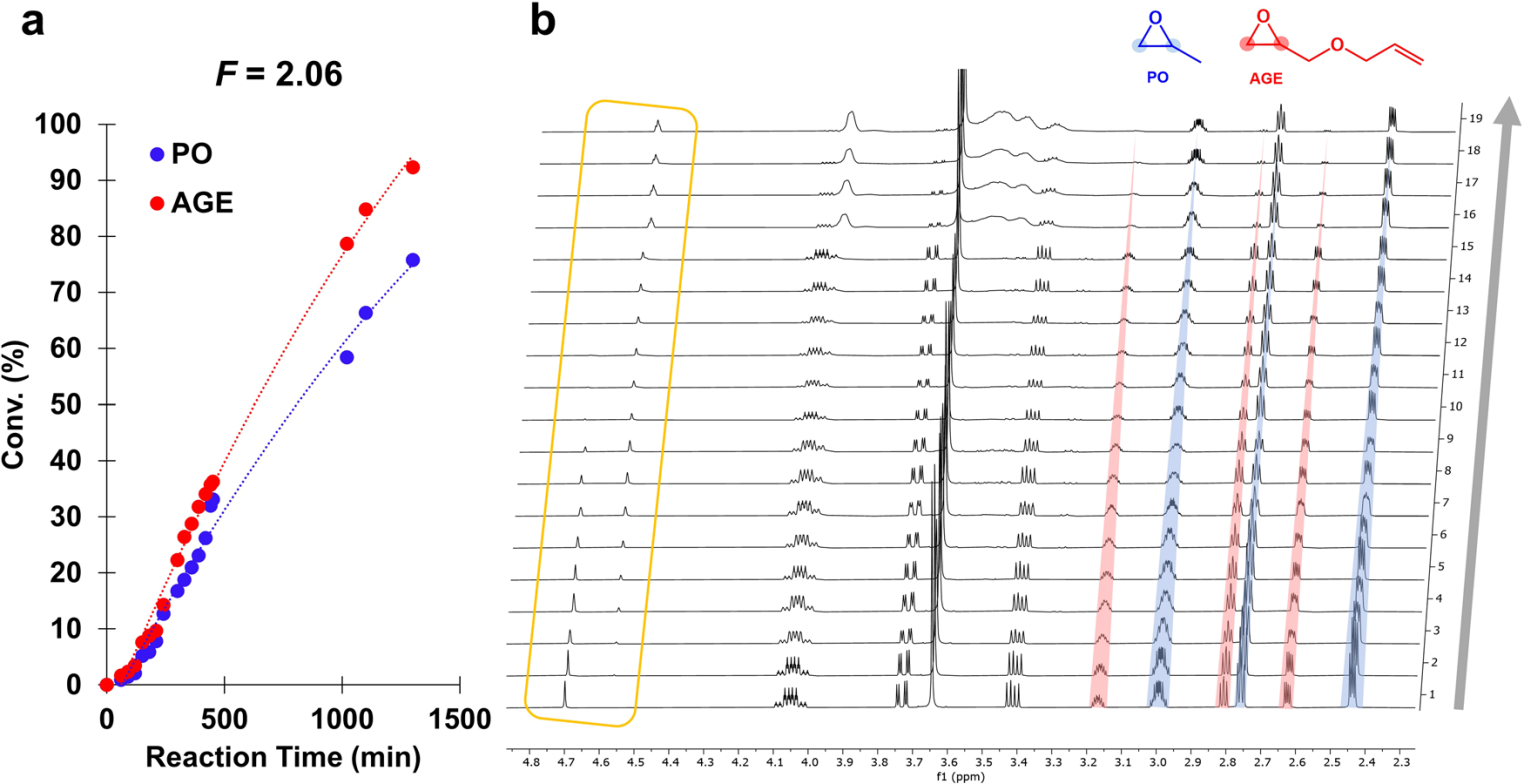
Kinetic profile of PO/AGE copolymerization. **a** Kinetic of PO and AGE copolymerization with BnOH activated by 18C6/KOAc complex with *F* of 2.06 and (**b**) full stacked ^1^H NMR spectra (CDCl_3_, 500 MHz) of P(PO-*co*-AGE) crude media showing the consumption of PO monomer (characteristic signals highlighted in blue) and AGE (characteristic signals highlighted in red) in function of time. The area marked in yellow shows the conversion of methylene benzylic group (4.69 ppm) into the α-phenyloxy copolymers end-group (4.56 ppm).

In all kinetic copolymerization experiments, regardless of the initial monomer composition used, in early stages of copolymerizations both comonomers are incorporated into the chain at almost the same frequency. Moreover, unlike the 3 days required for homopolymerizations, in copolymerizations a few hours are more than sufficient to achieve almost quantitative conversions of both comonomers, as evidenced from the conversion curves for both PO and AGE for a targeted *F* ([PO]_0_/[AGE]_0_) of 2.06 (Fig. 5a). Therefore, each of the propagating species prefers to add the other monomer rather than react with its own type of monomer and thus cross-over propagation takes place. For copolymerizations with *F* < 1 and *F* > 1, once the monomer present in low concentrations has been consumed, homopolymerization of unreacted oxirane follows to form block sequences. As observed from a representative ^1^H NMR data, methylene BnOH signals are continuously shifted during the whole inhibition time of the process, indicating that BnOH starts the initiation pathway (Fig. 5b). The resulting PO/AGE copolymers were analyzed by ^1^H and ^13^C NMR spectroscopy (Supplementary Figs. 11–20).

The findings were underpinned by the determination of the reactivity ratios for monomer pair PO/AGE according to the Fineman−Ross method at low comonomer conversion (<10–15%) and after freeing ourselves from the inhibition period and taking into account the few percent of comonomers that reacted during this period (Table 2).

**Table 2:** Calculated parameters for Fineman–Ross equation in PO/AGE copolymerizations from BnOH activated by 18C6/KOAc complex.

Entry	*F* = [PO]_0_/[AGE]_0_^a^	${{{{{{\boldsymbol{F}}}}}}}_{{{{{{\boldsymbol{PO}}}}}}}$^b^	PO conv. (%)^c^	AGE conv. (%)^c^	${{{{{{\boldsymbol{f}}}}}}}_{{{{{{\boldsymbol{PO}}}}}}}$^b^
1	0.1	0.07	6	2	0.19
2	0.2	0.16	7	2	0.52
3	0.31	0.24	12	3	0.52
4	0.75	0.43	5	3	0.54
5	2.06	0.68	5	7	0.69
6	5.06	0.86	8	19	0.71

^a^F = molar fraction of comonomers in the initial feed.

^b^${F}_{{PO}}$, the initial molar fraction in PO in the feed and ${f}_{{PO}}$ the molar fraction of PO in the as-obtained copolymer (determined by ^1^H NMR). Formulas used are detailed in Supplementary Equation (1).

^c^as determined by ^1^H NMR spectroscopy.

**Fig. 6:**
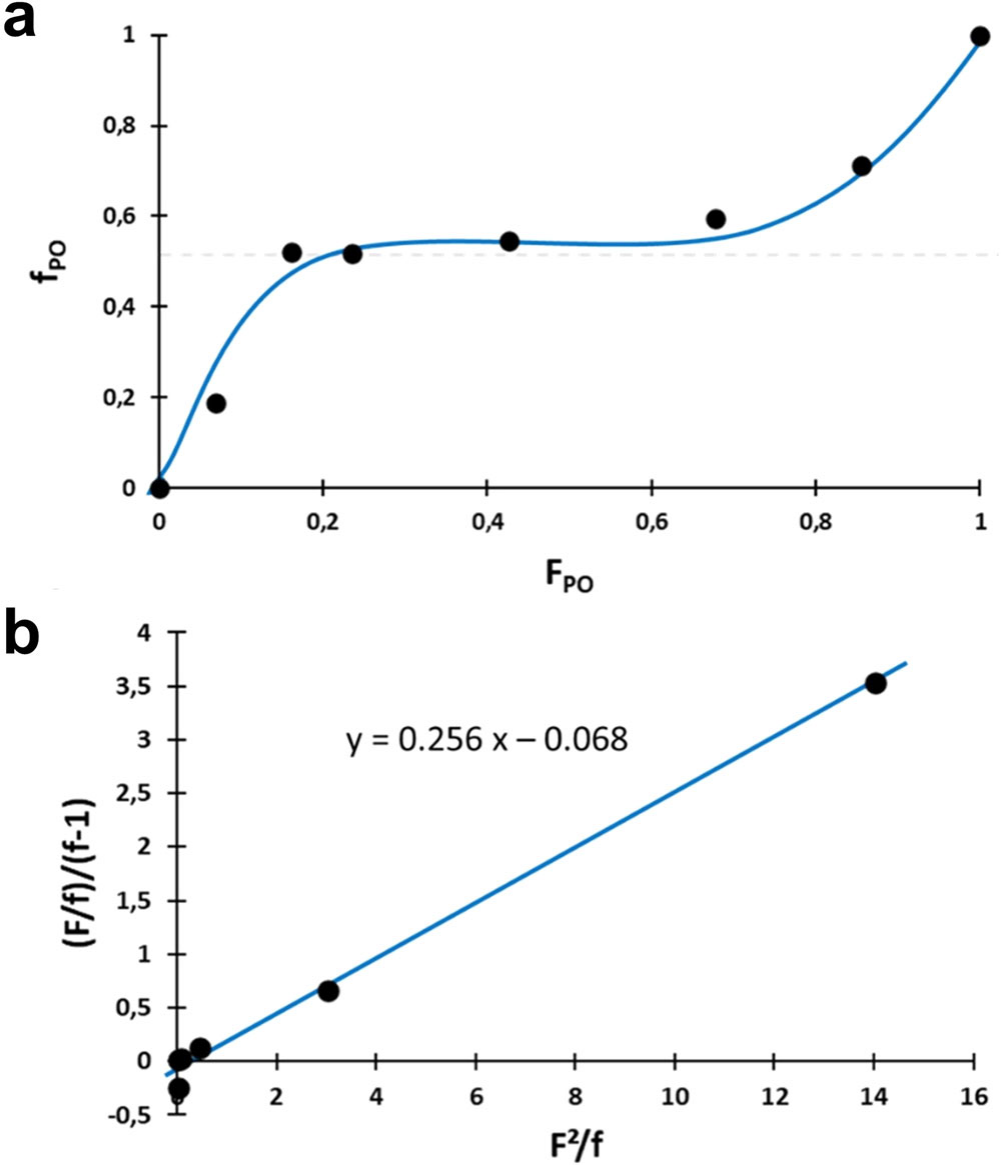
Fineman-Ross relationship for PO/AGE copolymerization. **a** Mole fraction of PO in the copolymer (${f}_{{PO}}$) plotted against its mole fraction in the feed (${F}_{{PO}}$). **b** Determination of reactivity ratios of PO and AGE using Fineman–Ross method.

As expected, the relation between the molar fraction of PO in the copolymer (${f}_{{PO}}$) and in the initial comonomer molar content (${F}_{{PO}}$) drawn from the kinetics, excluding the inhibition period, clearly indicates an alternating arragment of comonomers along the copolymer chain (Fig. 6a). The comonomer reactivity ratios calculated from the slope and *Y*-axis intercept were ${r}_{{PO}}$ = 0.25 and ${r}_{{AGE}}$ = 0.07, respectively (Fig. 6b). Although from these values of ${r}_{{PO}}$ and ${r}_{{AGE}}$ one can deduce that PO is slightly more reactive than AGE to go into the copolymer, which is possibly originated from the steric effect of the side chain, values of ${r}_{{PO}}$ and ${r}_{{AGE}}$ still quite close to zero imply rapid cross-propagation reactions, therefore, formation of partially alternating P(PO-*co*-AGE) copolymer is favored since the monomers show little inclination to homopolymerize. These results strongly support an quasi-alternating copolymerization profile.

### Comparison between quasi-alternating, gradient, and diblock copolymers of PO/AGE

In order to confirm, by comparison with other copolymer topologies, the microstructure of the quasi-alternating copolymers, we prepared PPO-*b*-PAGE diblock and gradient P(PO-*co*-AGE) copolymers. They were synthesized by chain extension reactions with successive feed additions of monomers at different reaction times, adapting a procedure reported by Lutz and coworkers^51,52^, to target a final DP ~ 25 and *F* = 1 ([PO + AGE]_0_/[BnOH]_0_/[18C6/KOAc]_0_ = 25/1/0.5)(Supplementary Table 2), so to have a precise and accurate comparison with the quasi-alternating copolymer.

**Fig. 7:**
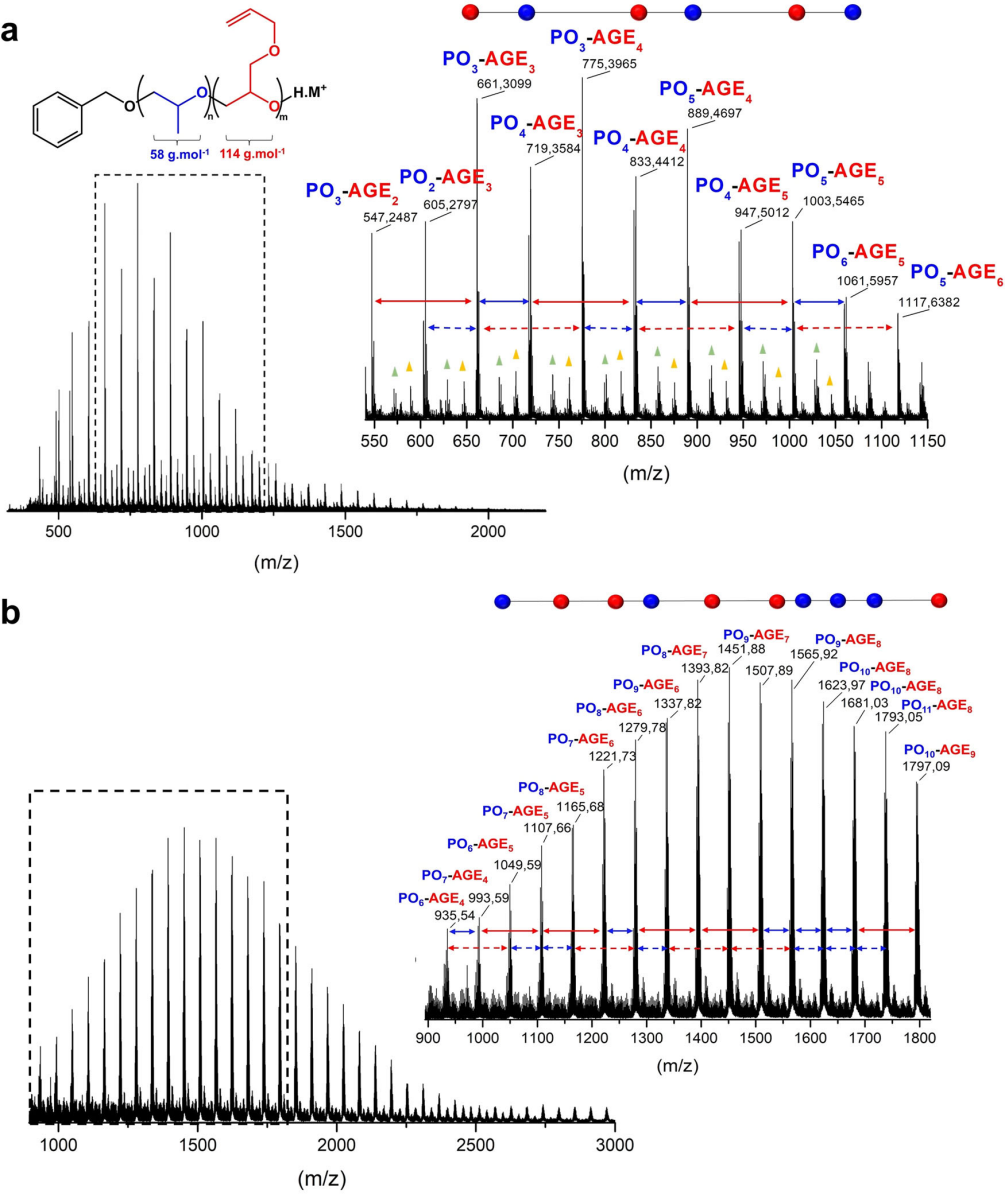
Fragmentation patterns of MALDI-ToF MS spectra of alternating *versus* gradient P(PO-*co*-AGE) copolymers. Full MALDI-ToF MS spectra of alternating P(PO-*co*-AGE) copolymer crude media after 18 h of reaction (**a**) and gradient P(PO-*co*-AGE) copolymer crude media after full conversion (**b**). Conditions: [PO + AGE]_0_/[BnOH]_0_ = 25 with *F* of 1. Polymer sample was dissolved in THF to obtain 1 mg.mL^−1^ solution. **a** The insets show the structure of the alternated copolymer (top left) and expanded view of the *m/z* range 550–1150, in which oligomers are observed (top right). All peaks correspond to C_7_H_8_OH(C_3_H_6_O)_n_(C_6_H_10_O_2_)_m_.K ^+^. The sign  designates H_2_O(C_3_H_6_O)_n_(C_6_H_10_O_2_)_m_.K ^+^. The sign  designates C_7_H_8_OH(C_3_H_6_O)_n_(C_6_H_10_O_2_)_m_.Na ^+^. **b** Expansion of the MALDI-ToF MS spectrum in the 900–1820 *m/z* region, showing the comonomer distribution in the range of fragments examined. All peaks correspond to C_7_H_8_OH(C_3_H_6_O)_n_(C_6_H_10_O_2_)_m_.Na^+^.

PO/AGE microstructure was first evaluated by matrix-assisted laser desorption/ionization time-of-flight mass spectrometry (MALDI-ToF MS). The monomodal peak distributions of MALDI-ToF MS spectra (Fig. 7, Supplementary Fig. 21) confirm the extent of control over the copolymerizations, the absence of undesired side reactions and, for diblock and gradient PO/AGE copolymers, as also evidenced by the evolution of SEC chromatograms recorded during the chain growth processes and the narrow associated dispersity values (*Ð*_*M*_ < 1.30, Supplementary Figs. 22–27). It has been previously shown that the fragmentation pattern of a MALDI-ToF MS spectrum of a copolymer can be directly related to its topology^53^, and indeed substantial differences in the fragmentation pathways could be found in our systems. In the MALDI-ToF MS analysis of the quasi-alternating P(PO-*co*-AGE) copolymer both comonomers are incorporated in the polymer chain and the PO and AGE units arrange in an alternating sequence when the feed ratio is 1:1 (Fig. 7a). The minor series could be assigned to complexes formed with water molecules present in the MALDI mixture, or to very minor initiation with water molecules. Importantly, no signals attributable to E2-based elimination fragments were observed. For gradient P(PO-*co*-AGE) copolymer, the pattern of peaks in the MALDI-ToF MS spectrum (Fig. 7b) appears different from that given by the alternating congener (Fig. 7a). The chemical distributions observed display an arrangement of the comonomers in a more random format. In contrast, along the chain of PPO-*b*-PAGE diblock copolymer, two distinct monomer blocks can be observed (Supplementary Fig. 21).

The distinctive elements found in copolymers MALDI-ToF MS spectra allowed the “mapping” of the chain composition, and served as a characteristic signature for each topology.

**Fig. 8:**
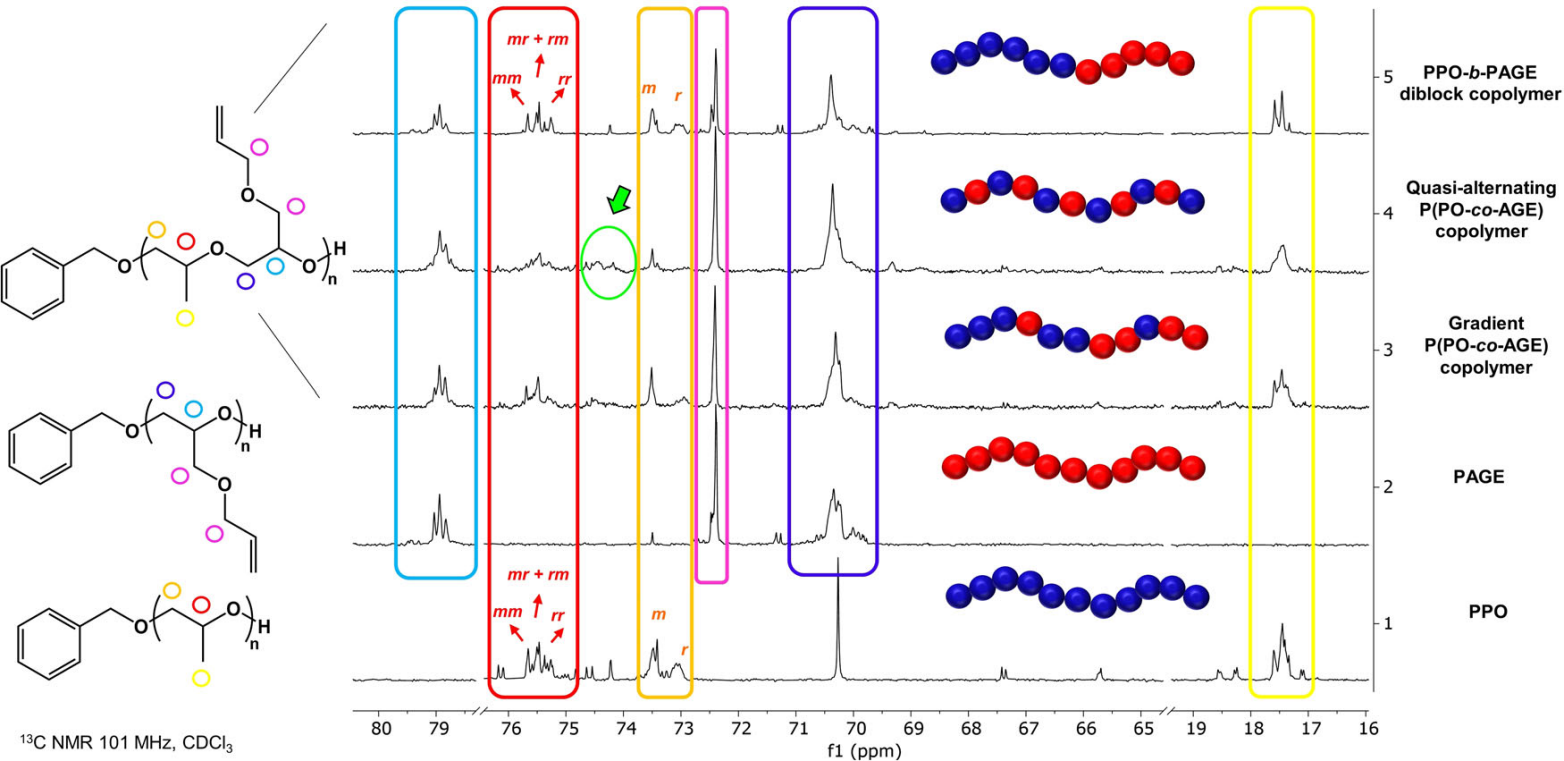
Comparative ^13^C NMR analyses for PPO, PAGE, and PO/AGE copolymers with different topologies. Comparison of ^13^C NMR spectra (CDCl_3_, 101 MHz) of the crude PPO, PAGE and the corresponding diblock, gradient and quasi-alternating PO/AGE copolymers with a focus on the methyl (characteristic signals highlighted in yellow), methine and methylene carbon regions (signals highlighted in red). The cross-peak region associated with mixed heterotriad sequences is tagged in green. The *m* and *r* refer to the meso and racemic.

^1^H and ^13^C NMR analyses of final PO/AGE copolymers are further evidence of the peculiar catalytic performance of the ROP system developed in controlling the copolymer architecture. (Supplementary Figs. 11–20). As reported by Lynd and coworkers^37^, the benzylic protons are sensitive to the sequence of the monomers added to the initiator and provide a record of the early events occurring during the copolymerization. In this respect, all ^1^H NMR spectra of the quasi-alternating copolymers provide consistent results indicating that the BnOH activated by 18C6/KOAc complex can indiscriminately attack both monomers, as demonstrated by the chemical shift values of the PhCH_2_ end-groups in the copolymers being intermediate to those of the two homopolymers (Supplementary Figs. 11–13, 15–16, 18, and 19). The benzyl proton resonances of resulting diblock and gradient copolymers match perfectly to those of homopolymers (Supplementary Figs. 23 and 26). ^13^C NMR spectra contain additional prominent cross-peaks from mixed PO/AGE heterotriad sequences as a result of neighboring PO and AGE repeat units, that are present at very low intensity in the case of gradient P(PO-*co*-AGE) copolymer, while total absent in the PPO-*b*-PAGE diblock copolymer, supporting a quasi-alternating-type copolymer microstructure (Fig. 8, Supplementary Figs. 14, 17, 20, 24, and 27). The ^13^C NMR spectrum of the diblock copolymer revealed sharp peaks associated with PPO and PAGE homotriad sequences (Supplementary Figs. 28 and 29).

## Conclusions

In conclusion, we thoroughly investigated the formation kinetics of PPO and PAGE homopolymers, as well as of P(PO-*co*-AGE) copolymers, synthesized from BnOH activated with 18C6/KOAc complex. The 18C6/KOAc complex is a really efficient and robust system to promote copolymerization of both oxirane monomers with no detrimental effect of side reactions. The reactivity ratios determined by ^1^H NMR kinetics, using Fineman-Ross method, and the structural information derived from a combination of analyses provide important understanding of the 18C6/KOAc-mediated copolymerization, which provided a remarkable “quasi-alternating” process. Furthermore, sequence-controlled diblock and gradient copolymers with tunable molecular weight and microstructure are readily achieved. Taken together, these results demonstrate the remarkable ability of a single acetate-based catalyst to build copolyethers with well-defined architectures and controlled sequences in response to the type of “monomer diet” in the reaction feed. To the best of our knowledge, we reported the first case in which two epoxides are arranged in a unique quasi-alternating architecture. The controlled copolymerization of oxiranes mediated by 18C6/KOAc complex opens up new avenues for programmable design of new alternating, eventually stereodefined, copolymer platforms providing control of mechanical-functional properties not accessible with the more common gradient or block copolymers. Our future studies will focus on how these differences in tacticity affect the physical and chemical properties of such copolymers to guide the development of new materials for functional applications.

## Experimental

### Materials and Methods

#### Materials

Propylene oxide (PO, ≥99%, Aldrich) and allyl glycidyl ether (AGE, ≥99%, Aldrich) were dried over CaH_2_, distilled and stored over molecular sieves. Benzyl alcohol (BnOH) and isopropyl alcohol (iPrOH) were dried over CaH_2_ for 48 h prior to their distillation under reduced pressure. Potassium acetate (KOAc, ≥99%, VWR) was dried by heating at 100 °C under vacuum for 48 h. 18-Crown ether-6 (18C6, 99%, ACROS Organics) was dried by three azeotropic distillations of tetrahydrofuran (THF). Compounds were all stored in a glove box (O_2_ ≤ 6 ppm, H_2_O ≤ 1 ppm). THF solvent was dried using a MBraun Solvent Purification System (model MB-SPS 800) equipped with alumina drying columns.

#### General techniques

^1^H and ^13^C nuclear magnetic resonance (NMR) spectra were recorded using a Bruker AVANCEII 500 MHz apparatus and a Bruker Avance III 400 MHz spectrometer at room temperature in chloroform-*d*_1_ (CDCl_3_). All spectra were acquired according to the following parameters: aq = 6.8 s, d1 = 10 s, sw = 12, o1p = 5, ns = 128 (4000 for ^13^C), PULPROG zg30. Size exclusion chromatography (SEC) was performed in THF at 35 °C using a Triple Detection Polymer Laboratories liquid chromatograph equipped with a refractive index (ERMA 7517), a UV detector (254 nm), a capillary viscometry, a light scattering RALS (Viscotek T-60) (Polymer Laboratories GPC-RI/CV / RALS) and an automatic injector (Polymer Laboratories GPC-RI/UV) and four columns : a PL gel 10 µm guard column and three PL gel Mixed-B 10 µm columns (linear columns for separation of M_w_PS ranging from 500 to 10^6^ daltons). All samples for SEC analyses were prepared according to the following concentration: 5–10 mg of polymer sample in 1 mL of THF. Positive-ion MALDI-Mass Spectrometry (MALDI-MS) experiments were recorded using a Waters QToF Premier mass spectrometer equipped with a Nd:YAG (third harmonic) operating at 355 nm with a maximum output of 65 µJ delivered to the sample in 2.2 ns pulses at 50 Hz repeating rate. Time-of-flight mass analyses were performed in the reflectron mode at a resolution of about 10,000. All the samples were analyzed using trans-2-[3-(4-tert-butylphenyl)-2-methylprop-2-enylidene]malononitrile (DCTB) as matrix. That matrix was prepared as 40 mg.mL^−1^ solution in CHCl_3_. The matrix solution (1 μL) was applied to a stainless steel target and air-dried. Polymer samples were dissolved in THF to obtain 1 mg.mL^−1^ solutions. Therefore, 1 μL of this solution was applied onto the target area already bearing the matrix crystals, and air-dried. For the recording of the single-stage MS spectra, the quadrupole (rf-only mode) was set to pass all the ions of the distribution, and they were transmitted into the pusher region of the time-of-flight analyzer where they were mass analyzed with 1 s integration time. Data were acquired in continuum mode until acceptable averaged data were obtained. Molecular mechanics and molecular dynamics simulations have been performed with the COMPASSIII force field as implemented in the BIOVIA Materials Studio 2022 package. In practice, adducts and the 18C6/KOAc complex have been put in contact and then subjected to a geometry optimization followed by successive 5-ns long quenched dynamics (NVT; quench every 5 ps) at increasing temperature (T = 300 K, 400 K, 600 K and 1000 K). At each temperature, multiple quenched dynamics runs have been performed until energy convergence between two successive runs is reached. The impact of the presence of the initiator on the interactions between the propagating monomer and the acetate has been evaluated on one hand from the distance measurements between the hydrogen of the hydroxyl group of the propagating monomer and the oxygen of the acetate and on the other hand from the amplitude of the H-Bond interactions as implemented in the Dreiding forcefield. To compute interaction energies among pure PO and AGE homo-dimers, as well as PO/AGE hetero-dimers, each system have been first built and optimized at the COMPASSIII level followed by successive 5-ns long quenched dynamics (NVT; quench every 5 ps) at increasing temperature (T = 100 K, 200 K, 300 K and 400 K). At each temperature, multiple quenched dynamics simulations were performed until achieving energy convergence between consecutive runs. The interaction energies were then calculated as the energy difference between the energy of the dimers and the cumulative energy of its isolated components.

### Synthesis and characterization

#### General procedure for PO homopolymerization from BnOH

In a glove box, a vial equipped with a stir bar was charged with BnOH (21 mg, 0.194 × 10^−3^ mol), KOAc (9.4 mg, 0.096 × 10^−3^ mol), 18C6 (25.5 mg, 0.096 × 10^−3^ mol), and PO (281 mg, 4.8 × 10^−3^ mol). The reaction is then performed at 21 °C and kinetically studied by SEC analysis and ^1^H NMR spectroscopy.

^1^H NMR (CDCl_3_, 400 MHz): δ 1.07–1.14 (m, −CH_3_), 3.32–3.60 (broad m, −O − CH_2_ − CH(CH_3_) − O − ), 4.54 (s, Ph−CH_2_ − O − ), 7.17–7.33 (broad m, Ph−CH_2_ − O − ). ^13^C NMR (CDCl_3_, 101 MHz): δ 17.45 ( − CH_3_), 73.09 ( − O − CH_2_ − CH(CH_3_) − O − , rrm or mrr), 73.42 ( − O − CH_2_ − CH(CH_3_) − O − , m), 75.23 ( − O − CH_2_ − CH(CH_3_) − O − , rr), 75.47 ( − O − CH_2_ − CH(CH_3_) − O − , mr + rm), 75.66 ( − O − CH_2_ − CH(CH_3_) − O − , mm), 127.65/127.60/128.46/138.59 (Ph−CH_2_ − O − ).

#### General procedure for AGE homopolymerization from BnOH

In a glove box, a vial equipped with a stir bar was charged with BnOH (21 mg, 0.194 × 10^−3^ mol), KOAc (9.4 mg, 0.096 × 10^−3^ mol), 18C6 (25.5 mg, 0.096 × 10^−3^ mol), and AGE (554 mg, 4.8 × 10^−3^ mol). The reaction is then performed at 21 °C and kinetically studied by SEC analysis and ^1^H NMR spectroscopy.

^1^H NMR (CDCl_3_, 400 MHz): δ 3.40–3.67 (broad m, −O − CH_2_ − CH(CH_2_ − O − CH_2_ − CH = CH_2_) − O − ), 3.97–3.98 (d, −O − CH_2_ − CH = CH_2_), 4.53 (s, −Ph−CH_2_ − O − ), 5.13–5.27 (doublet of doublets, −O − CH_2_ − CH = CH_2_), 5.84–5.89 (m, −O − CH_2_ − CH = CH_2_), 7.32 (broad m, Ph−CH_2_ − O − ). ^13^C NMR (CDCl_3_, 101 MHz): δ 69.92 ( − O − CH_2_ − CH(CH_2_ − O − CH_2_ − CH = CH_2_) − O − , rrm or mrr), 69.31–70.35 ( − O − CH_2_ − CH(CH_2_ − O − CH_2_ − CH = CH_2_) − O − , m), 72.39 ( − O − CH_2_ − CH = CH_2_), 78.94 ( − O − CH_2_ − CH(CH_2_ − O − CH_2_ − CH = CH_2_) − O − ), 116.85 ( − O − CH_2_ − CH = CH_2_), 127.69/127.71/128.48/138.46 (Ph−CH_2_ − O − ), 134.89 ( − O − CH_2_ − CH = CH_2_).

#### General procedure for PO S_N_2 reaction with BnOH

In a glove box, a vial equipped with a stir bar was charged with BnOH (100 mg, 0.92 × 10^−3^ mol), KOAc (45 mg, 0.46 × 10^−3^ mol), 18C6 (122 mg, 0.46 × 10^−3^ mol), and PO (107 mg, 1.84 × 10^−3^ mol). The reaction is then performed at 21 °C and kinetically studied by SEC analysis and ^1^H NMR spectroscopy.

^1^H NMR (CDCl_3_, 500 MHz): δ 1.07–1.14 (m, −CH_3_), 3.27–3.48 (m, −O − CH_2_ − CH(CH_3_) − O − ), 4.55 (s, Ph−CH_2_ − O − ), 7.33 (broad m, Ph−CH_2_ − O − ).

#### General procedure for copolymerization of PO and AGE from BnOH

In a glove box, a vial equipped with a stir bar was charged with BnOH, KOAc, 18C6, PO and AGE. All copolymerizations were carried out in bulk at r.t. by targeting a degree of polymerization (DP = [PO + AGE]_0_/[BnOH]_0_) of 25 and varying the initial monomer composition (*F* = [PO]_0_/[AGE]_0_) (Supplementary Table 1). The polymerization kinetics were determined by ^1^H NMR spectroscopy in function of time. The resulting copolymers were analyzed by ^1^H and ^13^C NMR spectroscopy. The number average molar mass (*M*_*n*_) and the dispersity (*Ɖ*_*M*_) were determined by SEC analysis.

^1^H NMR (CDCl_3_, 400 MHz): δ 1.12 (m, −CH_3_), 3.40–3.70 (broad m, ( − O − CH_2_ − CH(CH_3_) − O− and −O − CH_2_ − CH(CH_2_ − O − CH_2_ − CH = CH_2_) − O − ), 3.99 (d, −O − CH_2_ − CH = CH_2_), 4.53–4.54 (s, −Ph−CH_2_ − O − ), 5.13–5.27 (doublet of doublets, −O − CH_2_ − CH = CH_2_), 5.84–5.89 (m, −O − CH_2_ − CH = CH_2_), 7.32 (broad m, Ph−CH_2_ − O − ). ^13^C NMR (CDCl_3_, 101 MHz): δ 17.46 ( − CH_3_), 69.33/70.36 ( − O − CH_2_ − CH(CH_2_ − O − CH_2_ − CH = CH_2_) − O − ), 72.40 ( − O − CH_2_ − CH = CH_2_), 73.50 ( − O − CH_2_ − CH(CH_3_) − O − ), 74.18/74.65/74.84 ( − O − CH_2_ − CH(R) − O − , PO/AGE heterotriads), 75.47 ( − O − CH_2_ − CH(CH_3_) − O − ), 78.94 ( − O − CH_2_ − CH(CH_2_ − O − CH_2_ − CH = CH_2_) − O − ), 116.84 ( − O − CH_2_ − CH = CH_2_), 127.66/127.70/128.47/138.47 (Ph−CH_2_ − O − ), 134.92 ( − O − CH_2_ − CH = CH_2_).

#### General procedure for sequential block copolymerization of PO and AGE

In a glove box, a vial equipped with a stir bar was charged with BnOH (23.9 mg, 0.22 × 10^−3^ mol), KOAc (10.8 mg, 0.11 × 10^−3^ mol), 18C6 (29 mg, 0.11 × 10^−3^ mol) and PO (170 mg, 2.9 × 10^−3^ mol). After complete monomer consumption of PO within 2 days, AGE (347 mg, 3 × 10^−3^ mol) was added, and the mixture was stirred at r.t. until full conversion was achieved. The number average molar mass (*M*_*n*_) and the dispersity (*Ɖ*_*M*_) were determined by SEC analysis at each stage of monomer incorporation. The resulting copolymer was analyzed by ^1^H and ^13^C NMR spectroscopy.

^1^H NMR (CDCl_3_, 400 MHz): δ 1.12 (m, −CH_3_), 3.40–3.70 (broad m, ( − O − CH_2_ − CH(CH_3_) − O− and −O − CH_2_ − CH(CH_2_ − O − CH_2_ − CH = CH_2_) − O − ), 3.99 (d, −O − CH_2_ − CH = CH_2_), 4.54 (s, −Ph−CH_2_ − O − ), 5.13-5.27 (doublet of doublets, −O − CH_2_ − CH = CH_2_), 5.84–5.89 (m, −O − CH_2_ − CH = CH_2_), 7.32 (broad m, Ph−CH_2_ − O − ). ^13^C NMR (CDCl_3_, 101 MHz): δ 17.02/17.27 ( − CH_3_), 69.69/70.28 ( − O − CH_2_ − CH(CH_2_ − O − CH_2_ − CH = CH_2_) − O − ), 72.12 ( − O − CH_2_ − CH = CH_2_), 73.11/72.78 ( − O − CH_2_ − CH(CH_3_) − O − ), 75.20 ( − O − CH_2_ − CH(CH_3_) − O − ), 78.63 ( − O − CH_2_ − CH(CH_2_ − O − CH_2_ − CH = CH_2_) − O − ), 116.55/116.97 ( − O − CH_2_ − CH = CH_2_), 127.34/127.38/128.15/138.27 (Ph−CH_2_ − O − ), 134.35/134.74 ( − O − CH_2_ − CH = CH_2_).

#### General procedure for sequence-controlled PO/AGE copolymerization

In a glove box, a vial equipped with a stir bar was charged with BnOH, KOAc, 18C6, and AGE to generate the first block. The synthesis was carried out in bulk at r.t. with successive feed additions of monomers. Each feed addition was carried out after monitoring by NMR full conversion of all monomers, which occurred after 48–72 h. The total quantities of monomers (vs. initiator) were calculated to target a final degree of polymerization (DP = [PO + AGE]_0_/[BnOH]_0_) of ~25 and *F* = [PO]_0_/[AGE]_0_ = 1 (Supplementary Table 2, Section 2.2). The number average molar mass (*M*_*n*_) and the dispersity (*Ɖ*_*M*_) were determined by SEC analysis during the entire chain growth process. The resulting copolymer was analyzed by ^1^H and ^13^C NMR spectroscopy.

^1^H NMR (CDCl_3_, 400 MHz): δ 1.12 (m, −CH_3_), 3.40–3.70 (broad m, ( − O − CH_2_ − CH(CH_3_) − O− and −O − CH_2_ − CH(CH_2_ − O − CH_2_ − CH = CH_2_) − O − ), 3.99 (d, −O − CH_2_ − CH = CH_2_), 4.53 (s, −Ph−CH_2_ − O − ), 5.13–5.27 (doublet of doublets, −O − CH_2_ − CH = CH_2_), 5.84–5.89 (m, −O − CH_2_ − CH = CH_2_), 7.32 (broad m, Ph−CH_2_ − O − ). ^13^C NMR (CDCl_3_, 101 MHz): δ 17.46/17.59 ( − CH_3_), 69.56/70.31 ( − O − CH_2_ − CH(CH_2_ − O − CH_2_ − CH = CH_2_) − O − ), 72.41/72.44 ( − O − CH_2_ − CH = CH_2_), 72.95/73.51 ( − O − CH_2_ − CH(CH_3_) − O − ), 74.55 (PO/AGE heterotriads), 75.48 ( − O − CH_2_ − CH(CH_3_) − O − ), 78.95 ( − O − CH_2_ − CH(CH_2_ − O − CH_2_ − CH = CH_2_) − O − ), 116.87/117.01 ( − O − CH_2_ − CH = CH_2_), 127.70/127.72/128.49/138.46 (Ph−CH_2_ − O − ), 134.91/135.05 ( − O − CH_2_ − CH = CH_2_).

### Supplementary information

Supplementary Information

Description of Additional Supplementary Files

Supplementary Data 1

## Data Availability

All processed data are available in the manuscript or supplementary information. Further NMR data are summarized in Supplementary Data 1.
