# Supplementary material for: Quasi-alternating copolymerization of oxiranes driven by a benign acetate-based catalyst
Source: Commun Chem. 2023 Oct 28;6:235. doi: 10.1038/s42004-023-01031-z (PMC10613202; doi:10.1038/s42004-023-01031-z)
Supplement: Supplementary file 1 — Supplementary Information [file 42004_2023_1031_MOESM1_ESM.pdf]

Supplementary Information

Quasi-Alternating Copolymerization of Oxiranes Driven by a Benign Acetate-based Catalyst

Charlotte Fornaciari[1,2], Vincent Lemaur[3], Dario Pasini[2]*, Olivier Coulembier[1]*

[1] Laboratory of Polymeric and Composite Materials (LPCM), Center of Innovation and Research in Materials and Polymers (CIRMAP), University of Mons, Place du Parc, 20, Mons 7000, Belgium.

[2] Department of Chemistry, University of Pavia, Viale Taramelli, 10, Pavia 27100, Italy.

[3] Laboratory for Chemistry of Novel Materials, Materials Research Institute, University of Mons, Place du Parc, 20, 7000 Mons, Belgium.

E-mails: olivier.coulembier@umons.ac.be

dario.pasini@unipv.it

Table of Contents

1. **Supplementary Tables**……………………………………………………..………S3

Supplementary Table 1. PO/AGE Copolymerizations....………...…………………..S3

Supplementary Table 2. Sequence-controlled Gradient Copolymerization….…….S5

2. **Supplementary Figures**…………………………………………………………..S6

2.1. Kinetics of PO and AGE Homopolymerizations from BnOH…........…..……S6

2.2. SEC traces of product obtained by PO S_N2 Reaction………………………..S7

2.3. ^{1}H spectrum of product between PO and BnOH………………....……....S8

2.4. ^{1}H and ^{13}C NMR spectra of PPO……………………………………..…...S9

2.5. ^{1}H and ^{13}C NMR spectra of PAGE……………………………….………..…S11

2.6. SEC traces of PO and AGE Homopolymerizations from iPrOH……...……S13

2.7. Overlay ^{1}H NMR spectra of PO and AGE mixtures…..……………………..S14

2.8. Kinetic Plots of PO and AGE Copolymerizations…….………..…………....S15

2.9. ^{1}H and ^{13}C NMR spectra of P(PO-*co*-AGE) Copolymers……………….…..S17

2.10. MALDI-ToF MS of PPO-*b*-PAGE Diblock Copolymer………..……………......S27

2.11. SEC, ^{1}H and ^{13}C NMR analyses of PPO-*b*-PAGE Diblock Copolymer……S28

2.12. SEC, ^{1}H and ^{13}C NMR analyses of Gradient P(PO-*co*-AGE) Copolymer...S31

2.13. Comparison ^{13}C NMR spectra of PO/AGE Copolymers……………………..S34

3. **Supplementary Equations**……………………….……...……….…...………S35

3.1. Fineman-Ross Copolymerization Equation……………...………….….……S35

4. **Supplementary References**…………………………………………………….S36

1. Supplementary Tables

Supplementary Table 1. PO/AGE Copolymerizations

F = 0.1

REAGENTS	eqv.	mol	amount
PO	2.3	0.44×10^{-3}	0.026 g
AGE	22.7	4.4×10^{-3}	0.502 g
BnOH	1	0.194×10^{-3}	0.021 g
KOAc	0.5	0.096×10^{-3}	0.0094 g
18C6	0.5	0.096×10^{-3}	0.0255 g

F = 0.2

REAGENTS	eqv.	mol	amount
PO	4.2	0.84×10^{-3}	0.049 g
AGE	20.8	4.2×10^{-3}	0.480 g
BnOH	1	0.2×10^{-3}	0.0219 g
KOAc	0.5	0.096×10^{-3}	0.0095 g
18C6	0.5	0.097×10^{-3}	0.0258 g

F = 0.31

REAGENTS	eqv.	mol	amount
PO	6	1.17×10^{-3}	0.068 g
AGE	19	3.7×10^{-3}	0.426 g
BnOH	1	0.194×10^{-3}	0.021 g
KOAc	0.5	0.096×10^{-3}	0.0094 g
18C6	0.5	0.096×10^{-3}	0.0256 g

***F* = 0.75**

REAGENTS	eqv.	mol	amount
PO	10.7	2.17×10^{-3}	0.126 g
AGE	14.3	2.9×10^{-3}	0.340 g
BnOH	1	0.2×10^{-3}	0.022 g
KOAc	0.5	0.096×10^{-3}	0.0095 g
18C6	0.5	0.096×10^{-3}	0.0255 g

***F* = 1**

REAGENTS	eqv.	mol	amount
PO	12.5	2.4×10^{-3}	0.142 g
AGE	12.5	2.4×10^{-3}	0.277 g
BnOH	1	0.194×10^{-3}	0.021 g
KOAc	0.5	0.096×10^{-3}	0.0095 g
18C6	0.5	0.096×10^{-3}	0.0254 g

***F* = 2.06**

REAGENTS	eqv.	mol	amount
PO	16.8	3.3×10^{-3}	0.196 g
AGE	8.2	1.6×10^{-3}	0.185 g
BnOH	1	0.196×10^{-3}	0.0213 g
KOAc	0.5	0.096×10^{-3}	0.0095 g
18C6	0.5	0.096×10^{-3}	0.0254 g

***F* = 5.06**

REAGENTS	eqv.	mol	amount
PO	21	4×10^{-3}	0.237 g
AGE	4	0.79×10^{-3}	0.091 g
BnOH	1	0.194×10^{-3}	0.021 g
KOAc	0.5	0.096×10^{-3}	0.0094 g
18C6	0.5	0.096×10^{-3}	0.255

Supplementary Table 2. Sequence-controlled Gradient PO/AGE Copolymerization

REAGENTS	Feed	eqv.	mol	Amount (g)
PO	1	-	-	-
	2	1	0.18×10^{-3}	0.0107
	3	4	0.55×10^{-3}	0.032
	4	5	0.74×10^{-3}	0.043
	5	3.5	0.63×10^{-3}	0.037
AGE	1	5	0.93×10^{-3}	0.106
	2	4	0.75×10^{-3}	0.086
	3	2	0.35×10^{-3}	0.040
	4	1	0.18×10^{-3}	0.021
	5	1	0.18×10^{-3}	0.021
BnOH	1	1	0.184×10^{-3}	0.020
KOAc	1	0.5	0.092×10^{-3}	0.009
18C6	1	0.5	0.092×10^{-3}	0.024

2. Supplementary Figures

2.1. Kinetics of PO and AGE Homopolymerizations with BnOH.

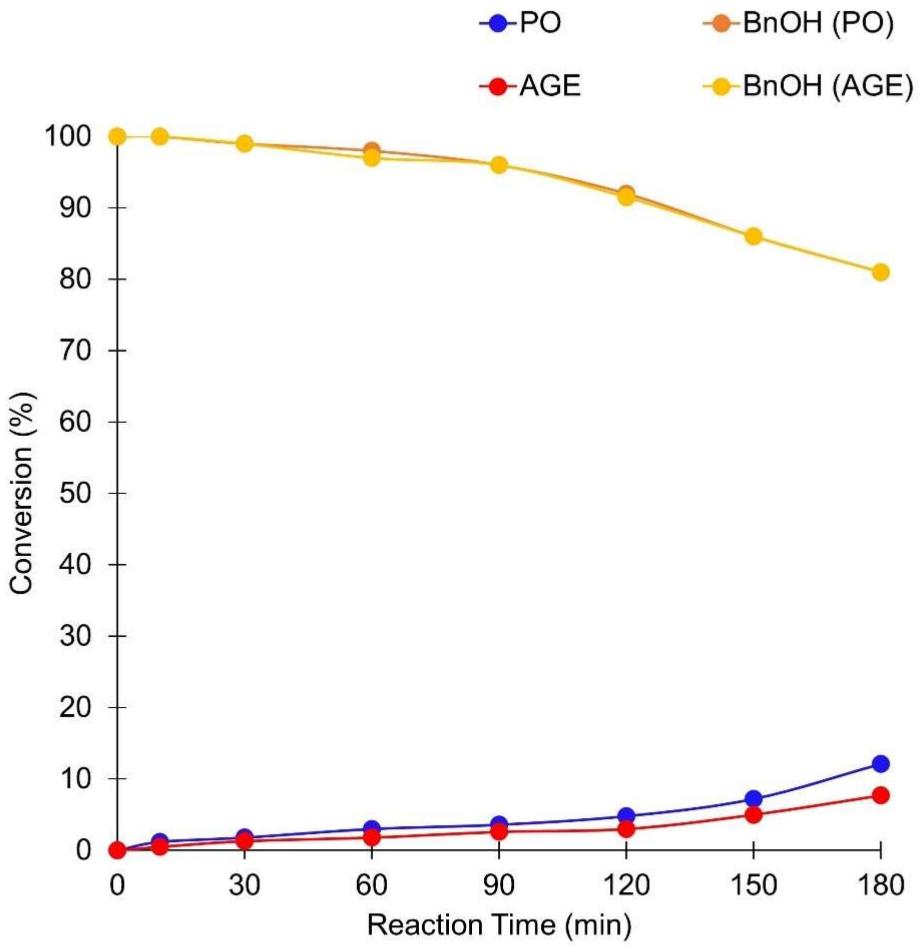

Supplementary Figure 1. Kinetics of PO and AGE homopolymerizations with BnOH activated by 18C6/KOAc complex. Conditions: $[M]_0/[BnOH]_0/[18C6/KOAc]_0 = 25/1/0.5$.

2.2. SEC traces of product obtained by PO S_N2 reaction.

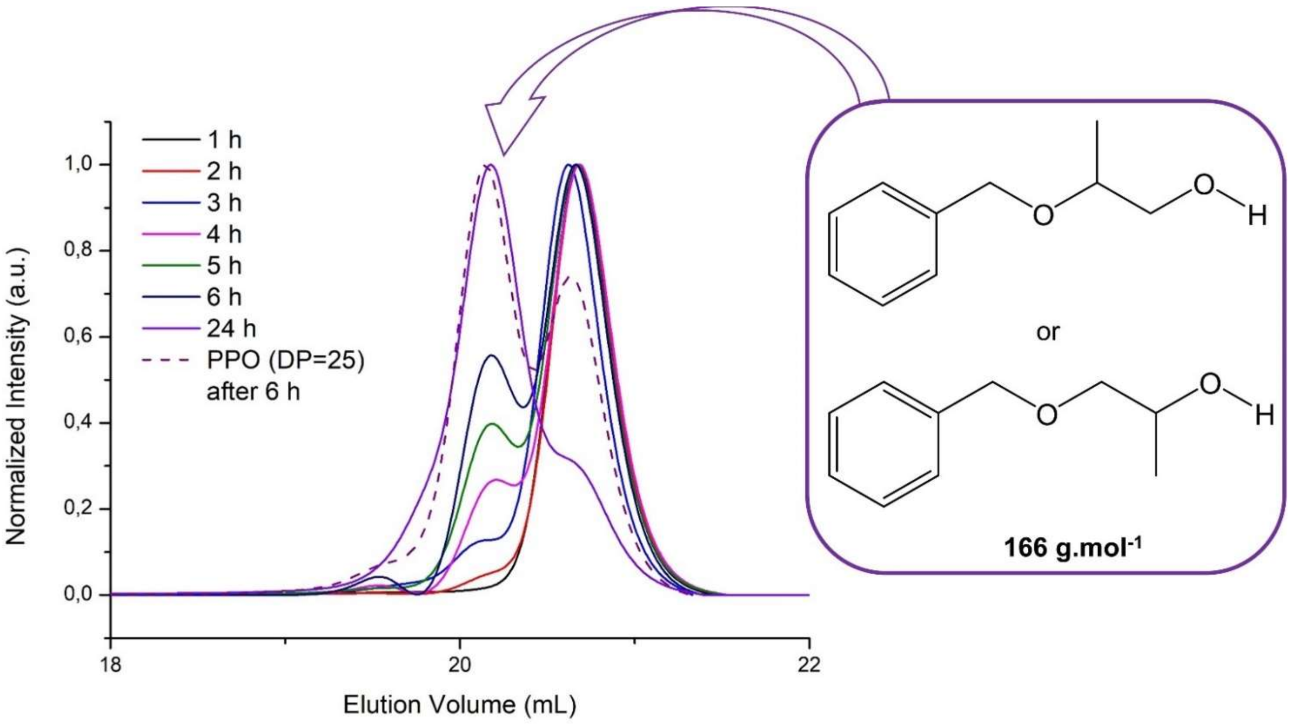

Supplementary Figure 2. Evolution of SEC traces of the crude product between PO and BnOH with the time. Conditions: $[PO]_0/[BnOH]_0/[18C6/KOAc]_0$ = 2/1/0.5.

2.3. ^{1}H NMR spectrum of product between PO and BnOH.

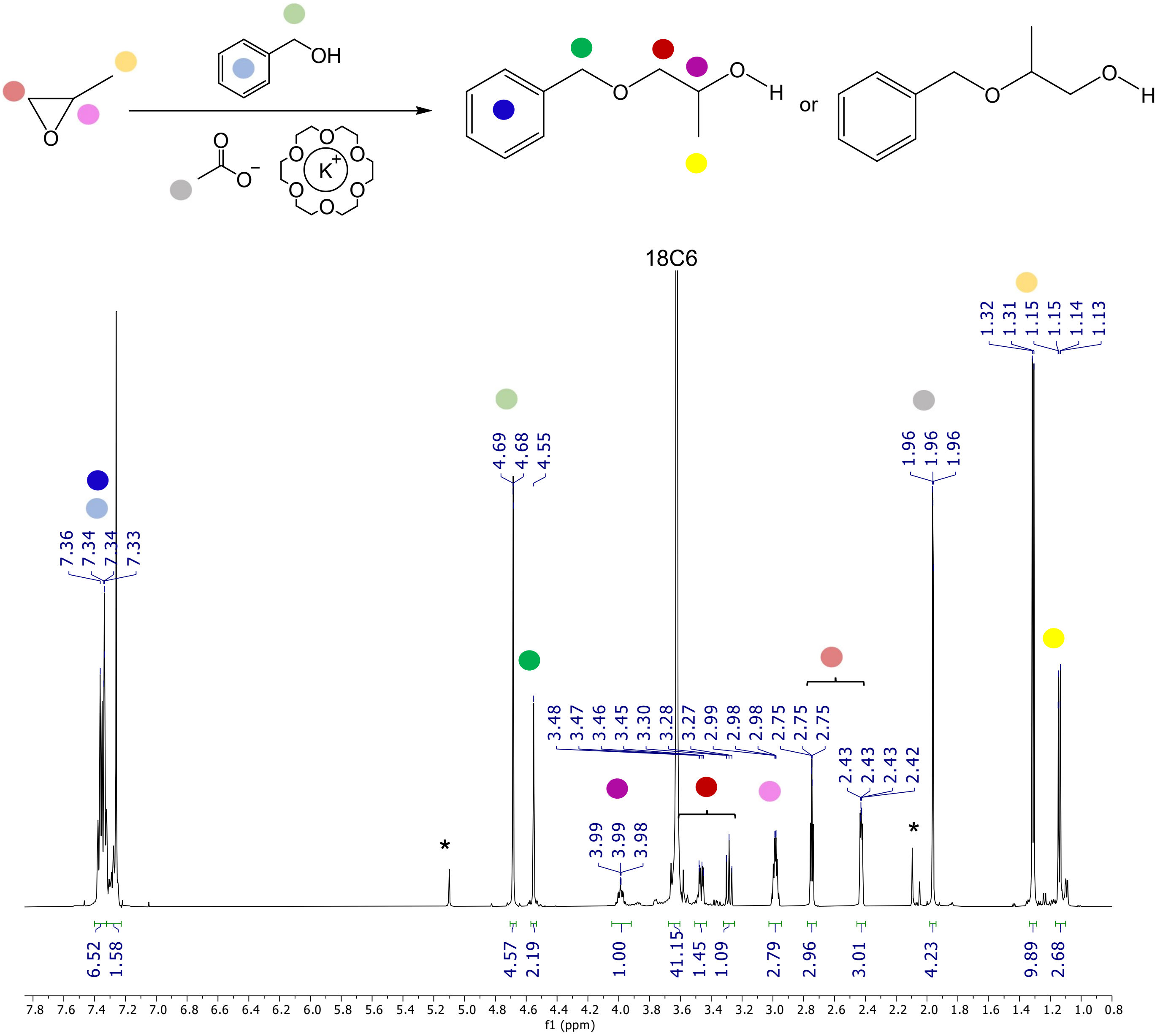

Supplementary Figure 3. ^{1}H NMR spectrum ($CDCl_3$, 500 MHz) of the crude product between BnOH and PO activated by 18C6/KOAc complex after 6 h. Condition: $[PO]_0/[BnOH]_0/[18C6/KOAc]_0$ = 2/1/0.5. (*) Signals associated to the presence of impurities in the crude products.

2.4. ^1H and ^{13}C NMR spectra of PPO.

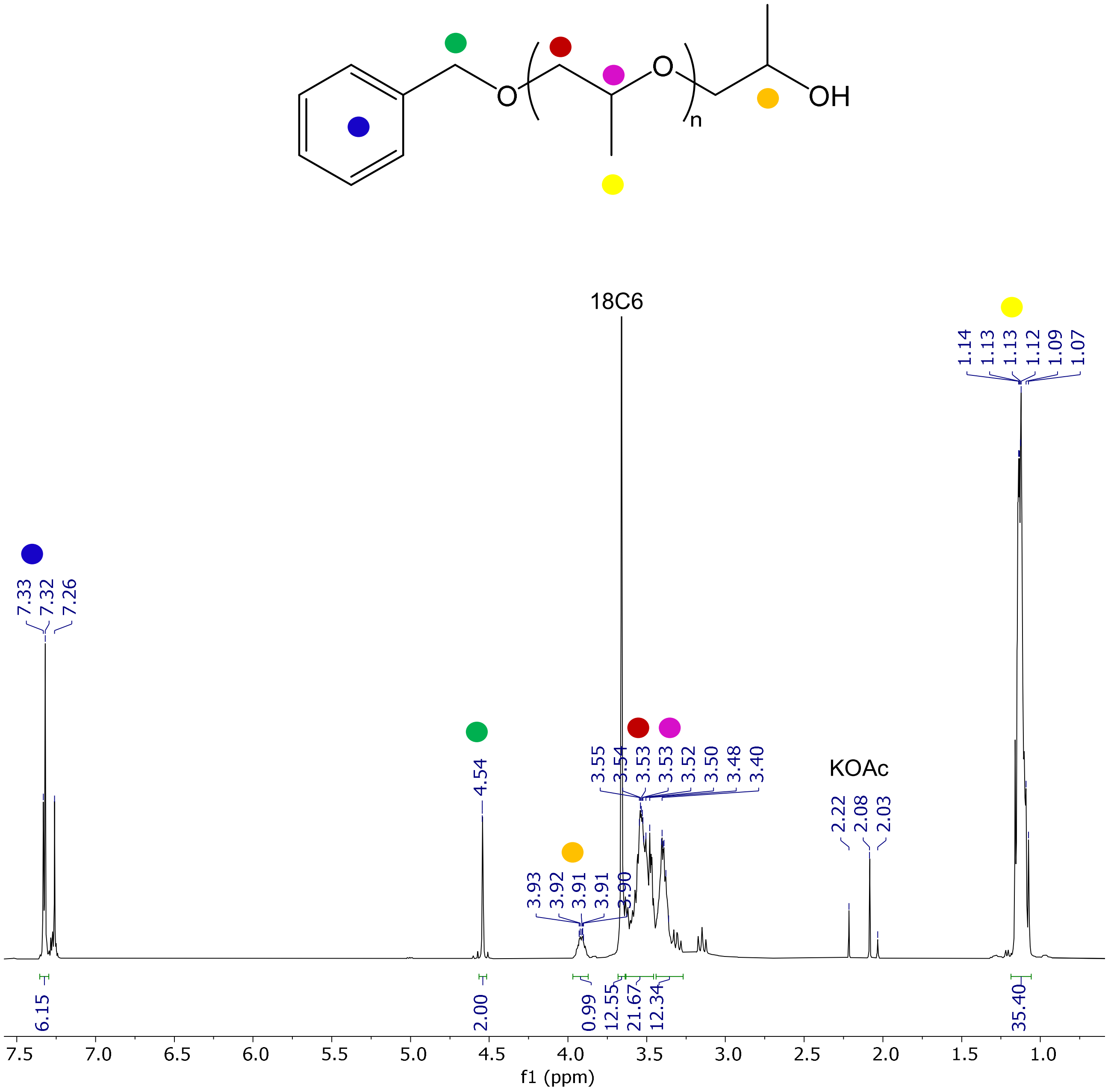

Supplementary Figure 4. ^1H NMR spectrum ($CDCl_3$, 400 MHz) of the crude PPO after full conversion. Condition: $[PO]_0/[BnOH]_0/[18C6/KOAc]_0$ = 25/1/0.5.

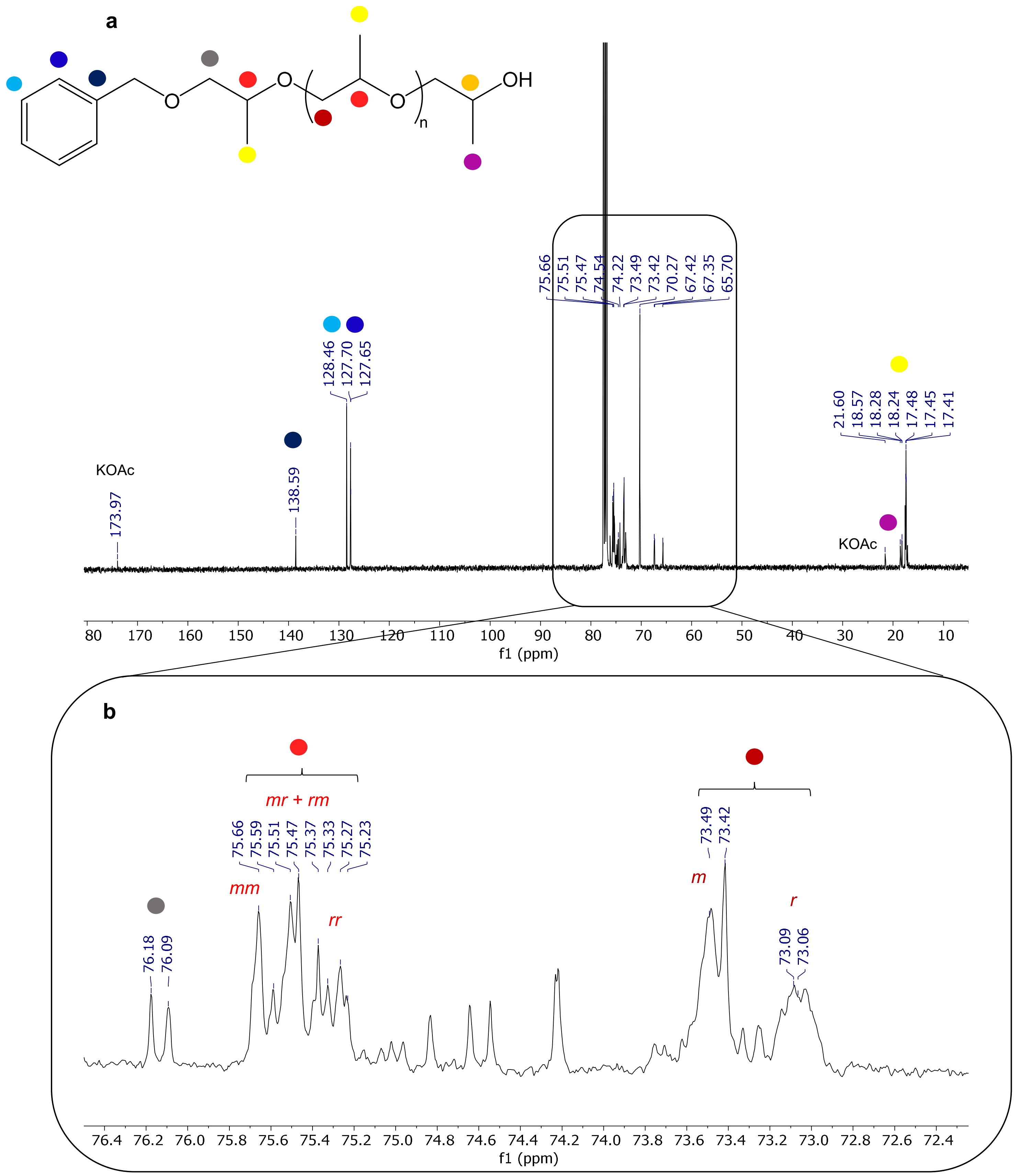

Supplementary Figure 5. (**a**) ^{13}C NMR spectrum ($CDCl_3$, 101 MHz) of the crude PPO (**b**) zoom of methine and methylene regions of atactic PPO. The *m* and *r* refer to the meso and racemic.

2.5. ^{1}H and ^{13}C NMR spectra of PAGE.

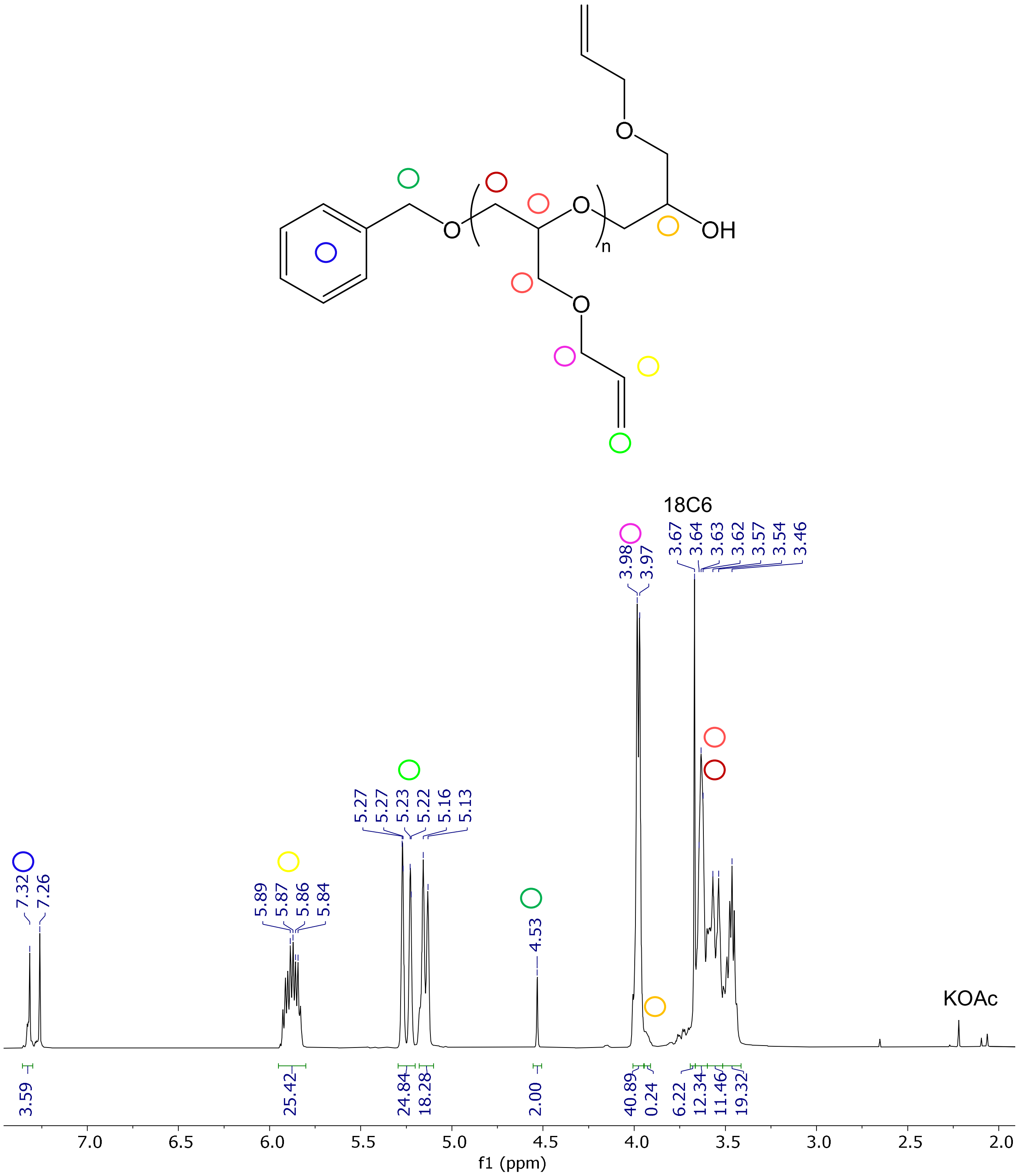

Supplementary Figure 6. ^{1}H NMR spectrum ($CDCl_3$, 400 MHz) of the crude PAGE after full conversion. Condition: $[AGE]_0/[BnOH]_0/[18C6/KOAc]_0$ = 25/1/0.5.

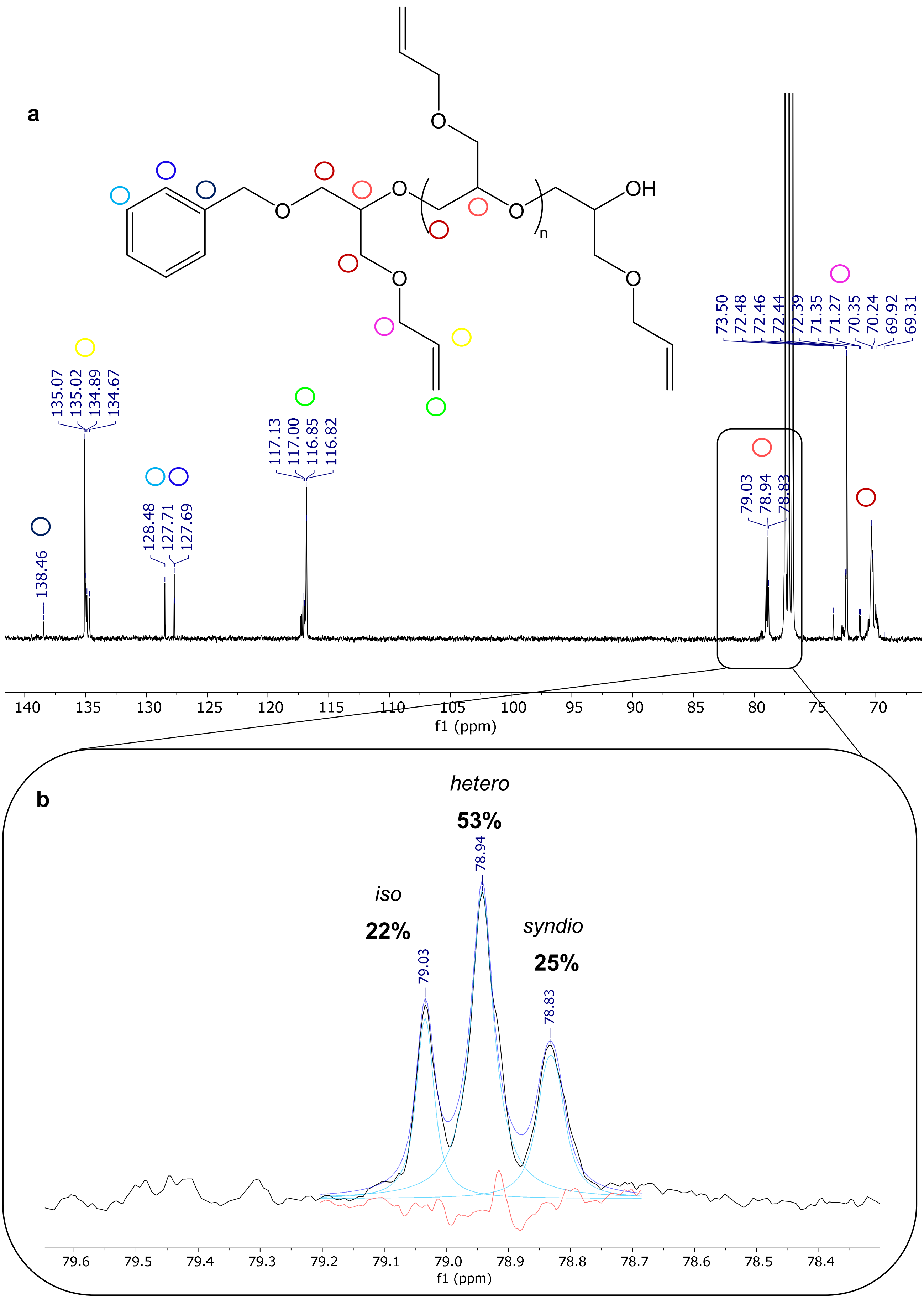

Supplementary Figure 7. (**a**) ^{13}C NMR spectrum ($CDCl_3$, 101 MHz) of the crude PAGE (**b**) with a focus on the region between 78.83 and 79.03 ppm that corresponds to the backbone methine carbon.

2.6. SEC traces of PO and AGE homopolymerizations from iPrOH.

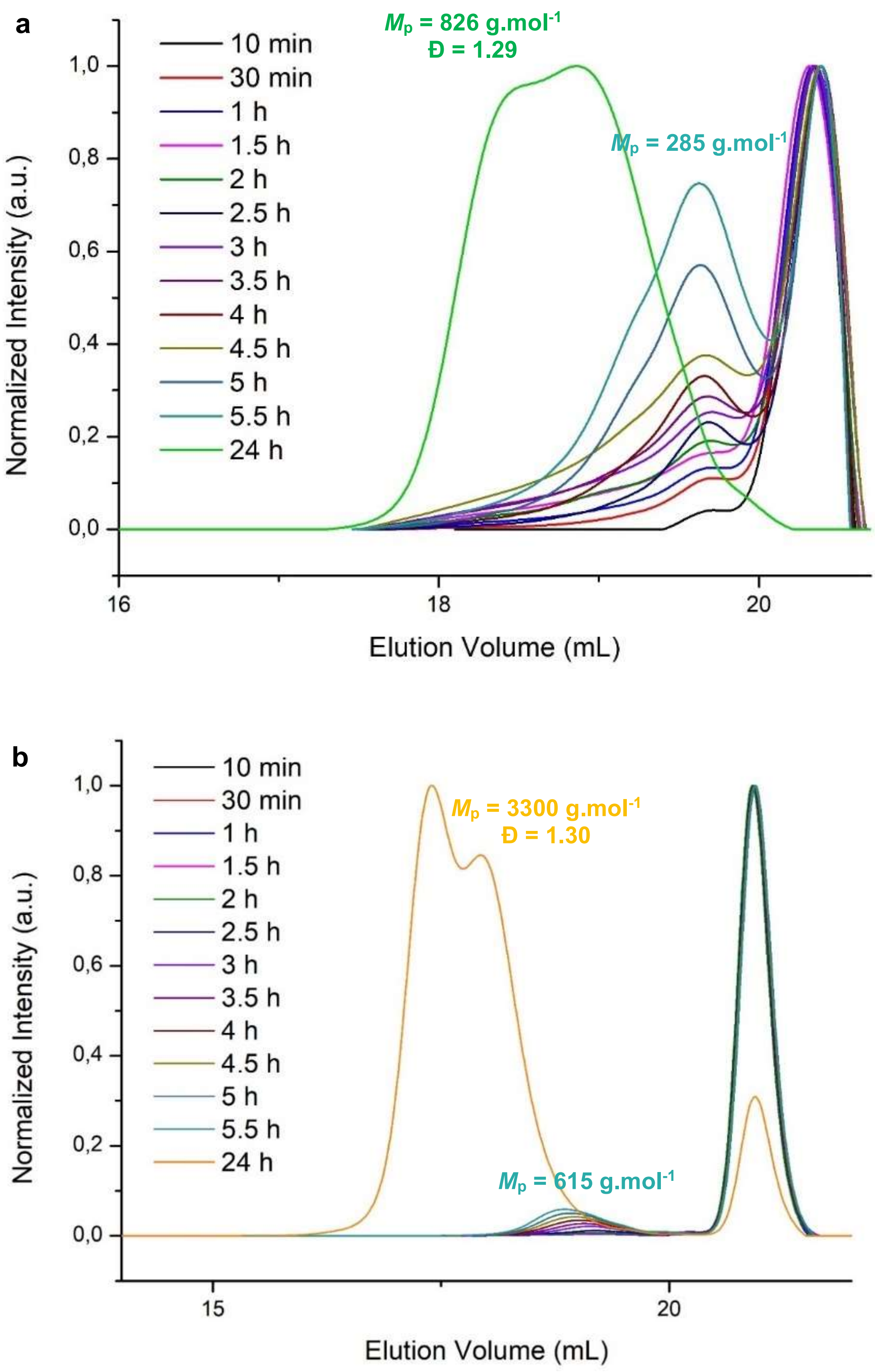

Supplementary Figure 8. Evolution of SEC traces in homopolymerizations of PO (**a**) and AGE (**b**) initiated with iPrOH. Conditions: $[M]_0/[iPrOH]_0/[18C6/KOAc]_0$ = 25/1/0.5.

2.7. Overlay ^{1}H NMR spectra of PO and AGE mixtures.

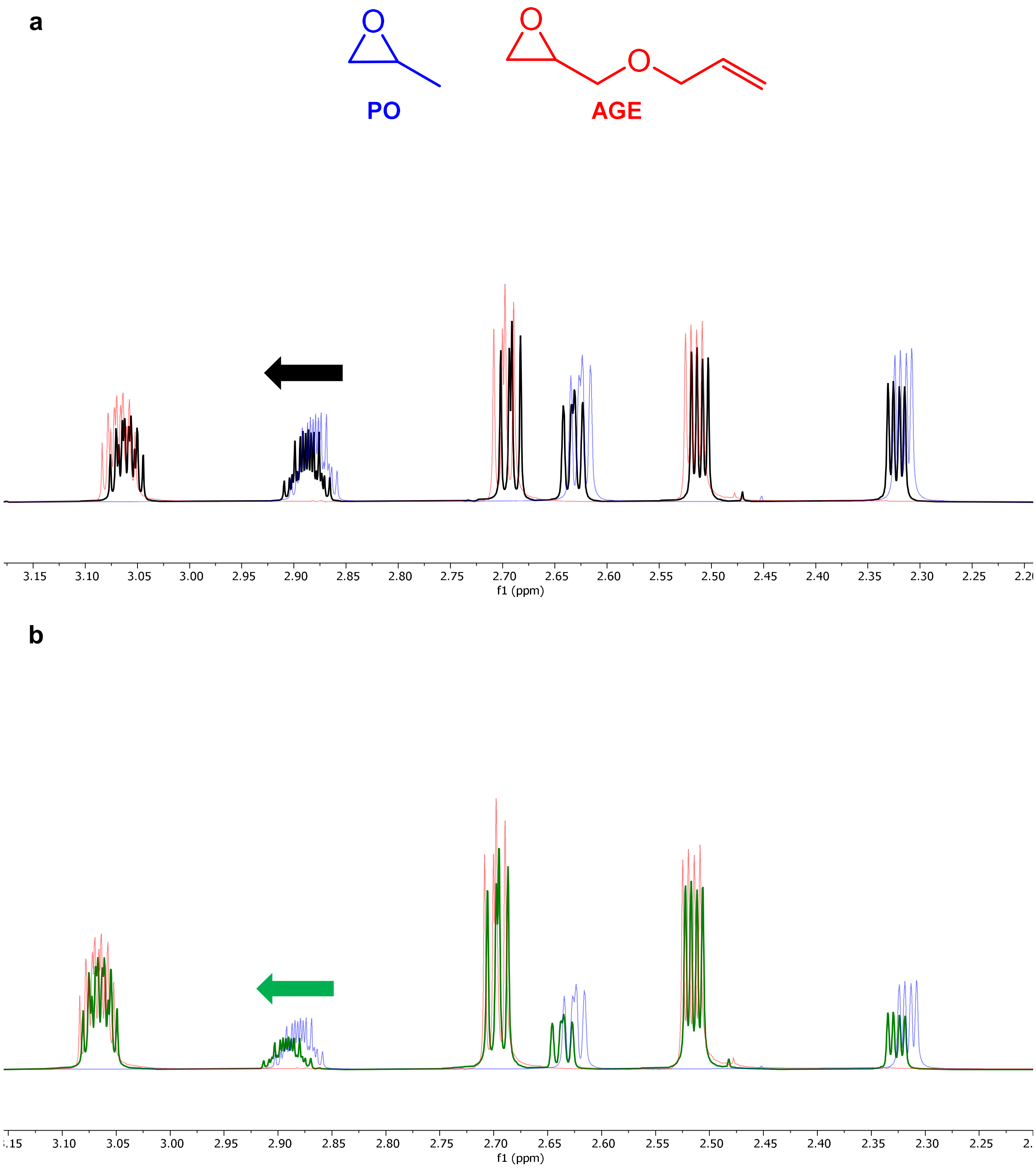

Supplementary Figure 9. Overlay ^{1}H NMR spectra (THF-d_8, 500 MHz) of several mixtures (**a**) $[PO]_0/[AGE]_0 = 1$ (black spectrum), (**b**) $[PO]_0/[AGE]_0 = 0.5$ (green spectrum) with PO (blue spectrum) and AGE (red spectrum). Total mixture concentrations of [PO], [AGE] and [PO+AGE] = 8.6 M in THF-d_8.

2.8. Kinetic plots of PO and AGE Copolymerizations.

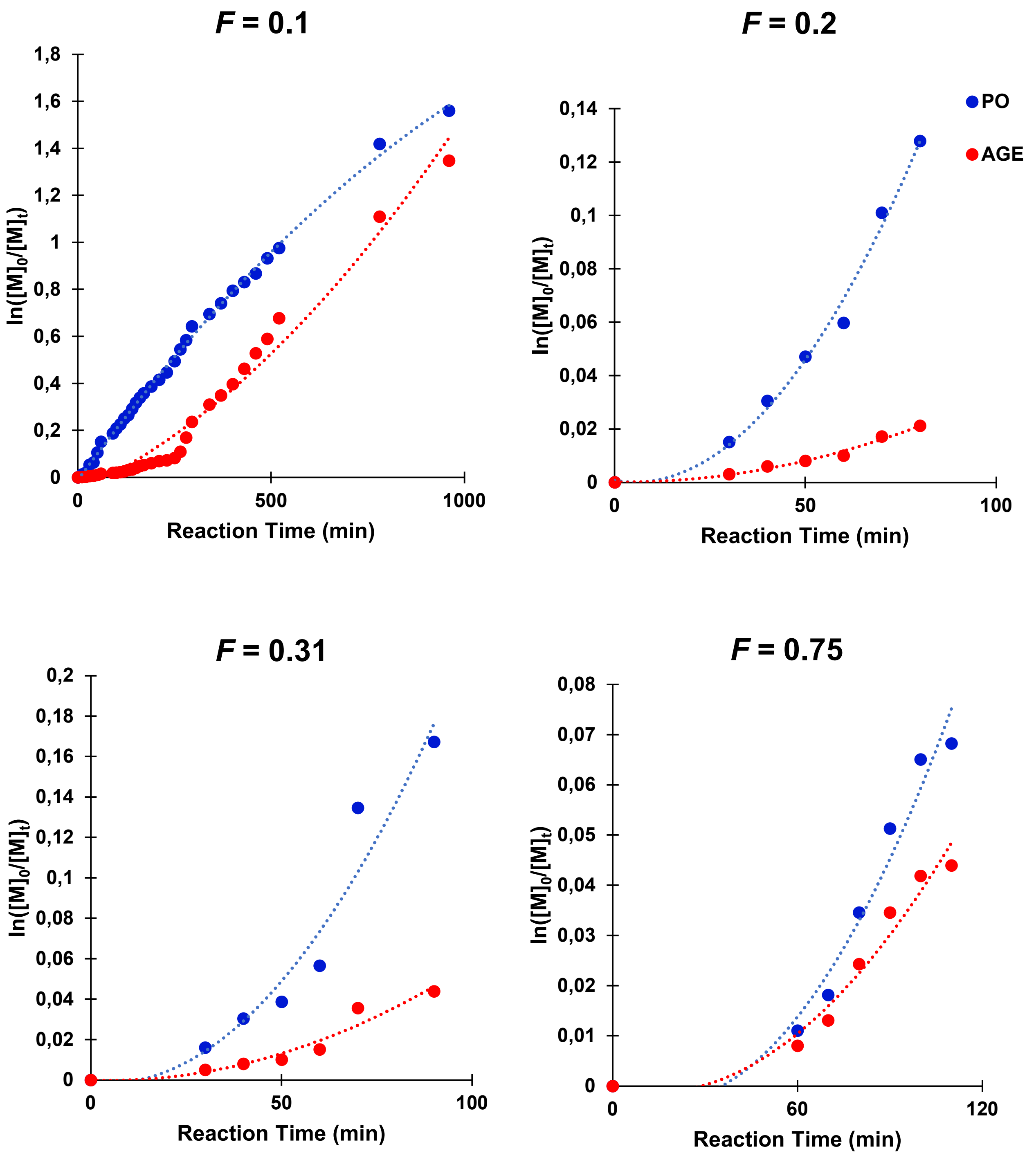

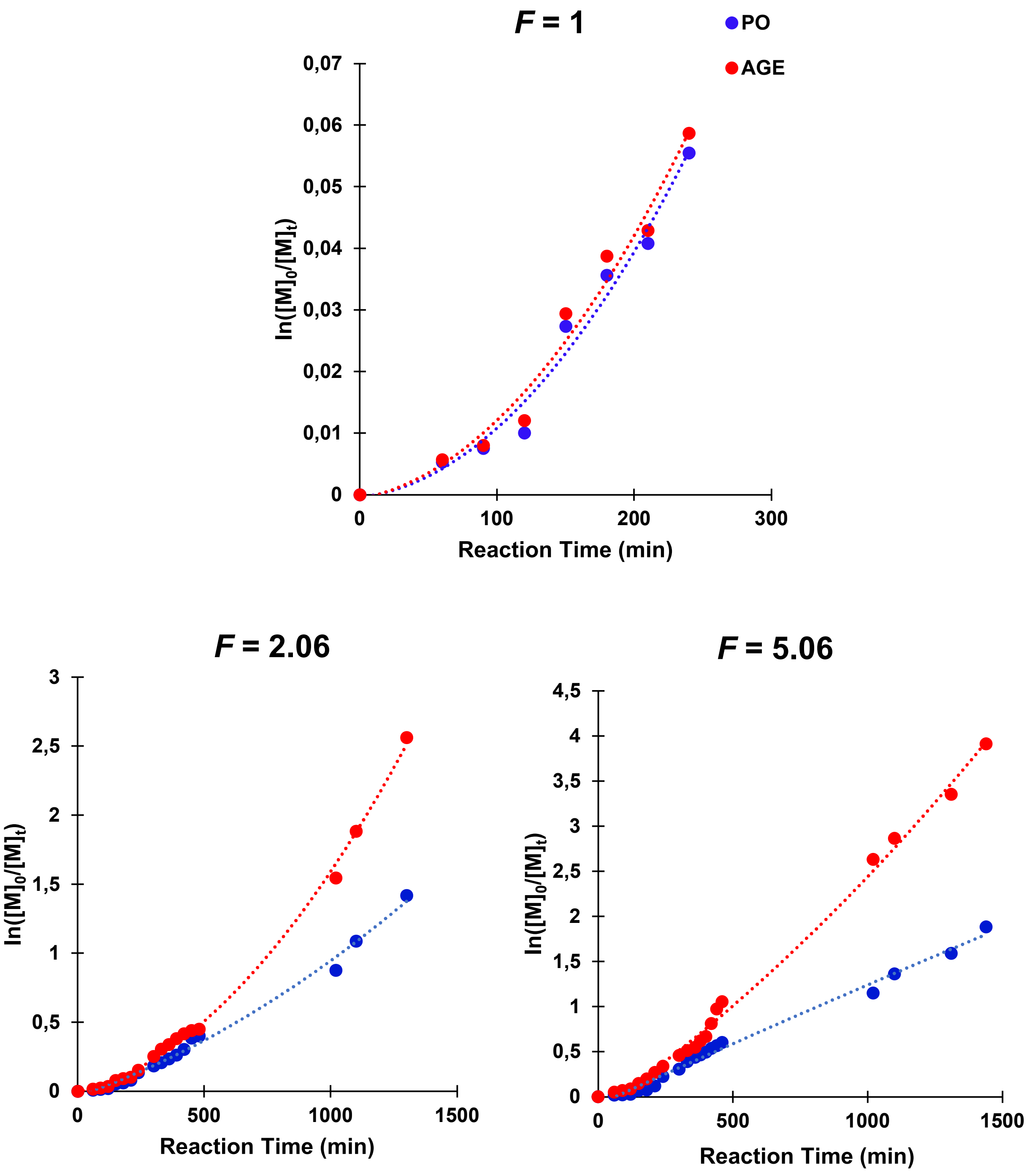

Supplementary Figure 10. Time dependence of the global conversion in the statistical copolymerization of PO and AGE at different molar fraction of PO. Associated semilogarithmic plots ($[M]_t$ measured by ^1H NMR spectroscopy, 500 MHz). The kinetic measurements were started after 30 minutes for $F < 1$, and after 60 minutes for $F > 1$, while each spectrum was recorded in a range of 10-20 minutes. Details of spectra acquisition: 10-15 mg of copolymer in 0.6 mL of $CDCl_3$, number of scans = 128.

2.9. ^{1}H and ^{13}C NMR spectra of P(PO-*co*-AGE) copolymers.

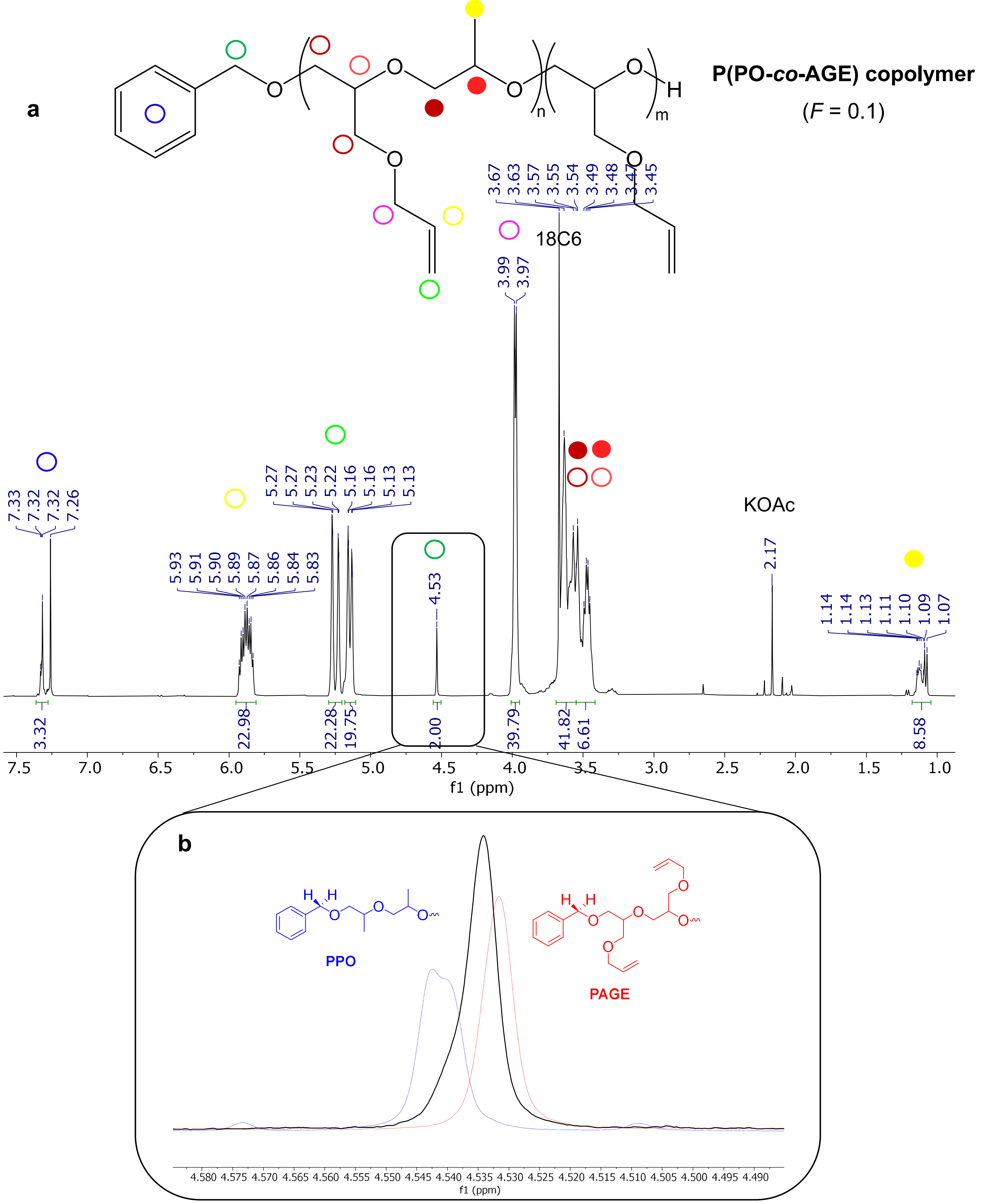

Supplementary Figure 11. (**a**) ^{1}H NMR spectrum ($CDCl_3$, 400 MHz) of P(PO-*co*-AGE) crude media. Conditions: $[PO+AGE]_0/[BnOH]_0 = 25$ with F of 0.1 after full conversion. (**b**) overlay ^{1}H NMR spectra to compare the α-phenyloxy end-groups of P(PO-*co*-AGE) (black spectrum) with the end-groups of PAGE (red spectrum) and PPO (blue spectrum).

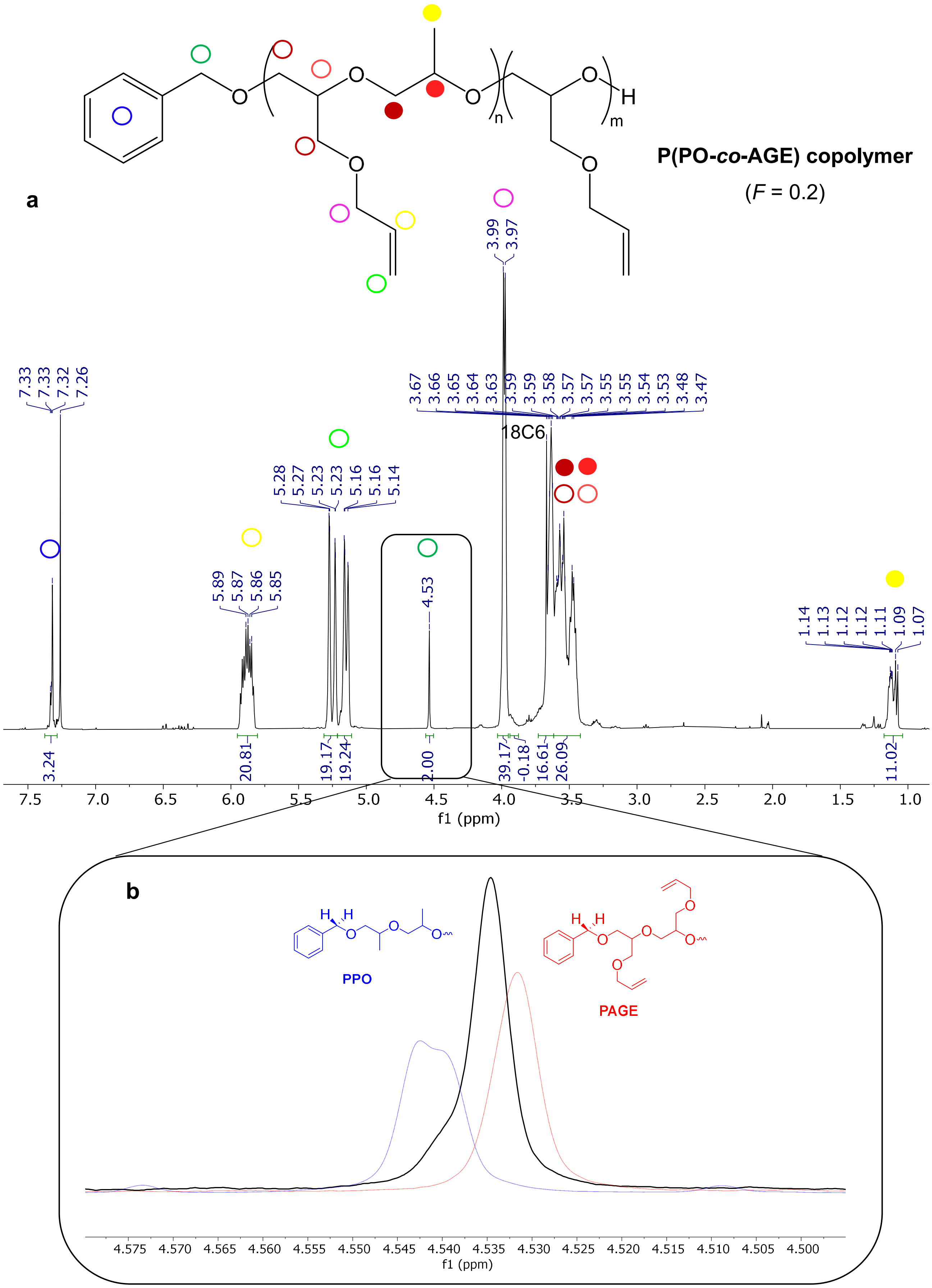

Supplementary Figure 12. (**a**) ^{1}H NMR spectrum ($CDCl_3$, 400 MHz) of P(PO-*co*-AGE) crude media. Conditions: $[PO+AGE]_0/[BnOH]_0$ = 25 with F of 0.2 after full conversion. (**b**) overlay ^{1}H NMR spectra to compare the α-phenyloxy end-groups of P(PO-*co*-AGE) (black spectrum) with the end-groups of PAGE (red spectrum) and PPO (blue spectrum).

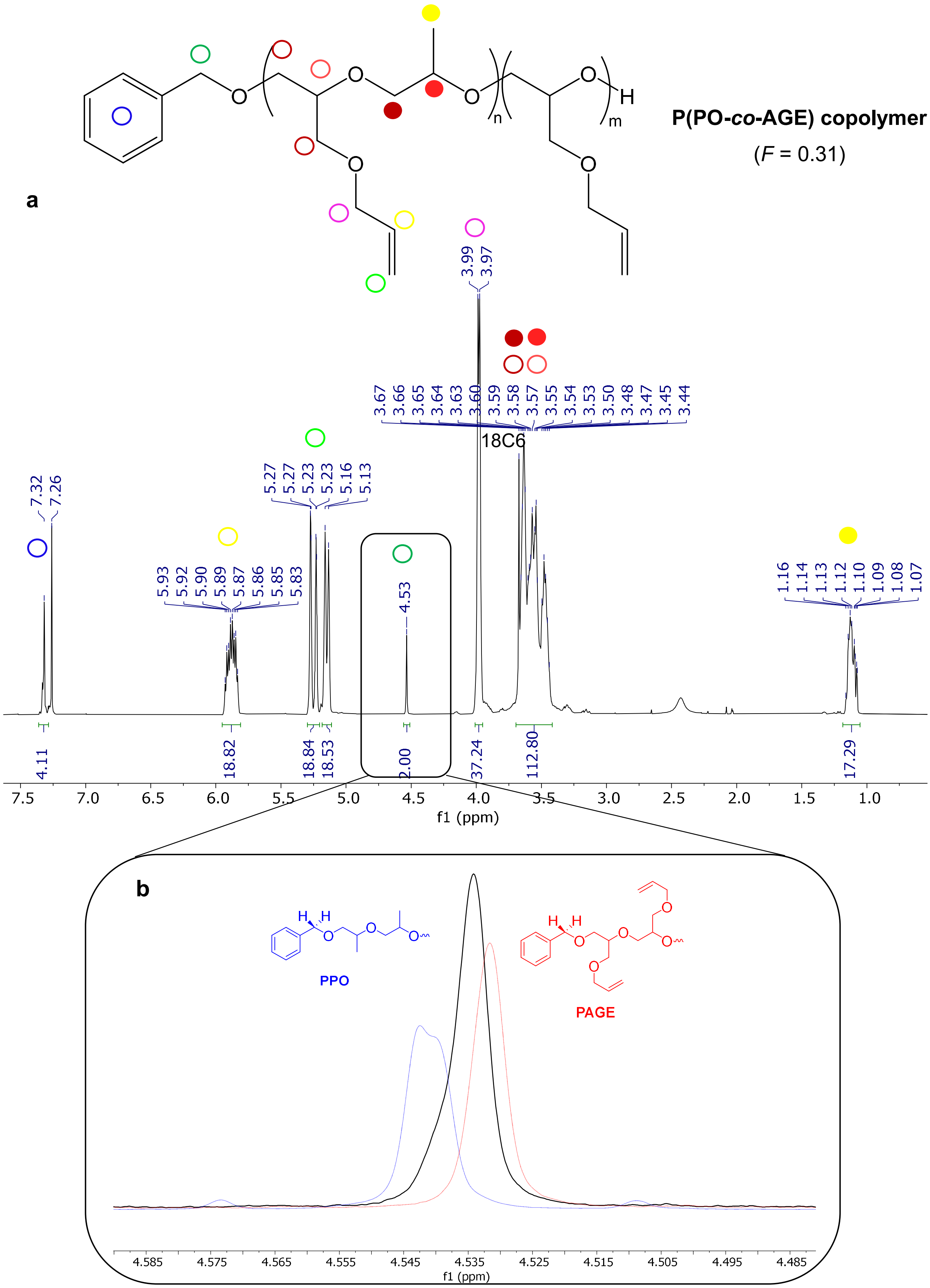

Supplementary Figure 13. (**a**) ^{1}H NMR spectrum ($CDCl_3$, 400 MHz) of P(PO-*co*-AGE) crude media. Conditions: $[PO+AGE]_0/[BnOH]_0$ = 25 with *F* of 0.31 after full conversion. (**b**) overlay ^{1}H NMR spectra to compare the α-phenyloxy end-groups of P(PO-*co*-AGE) (black spectrum) with the end-groups of PAGE (red spectrum) and PPO (blue spectrum).

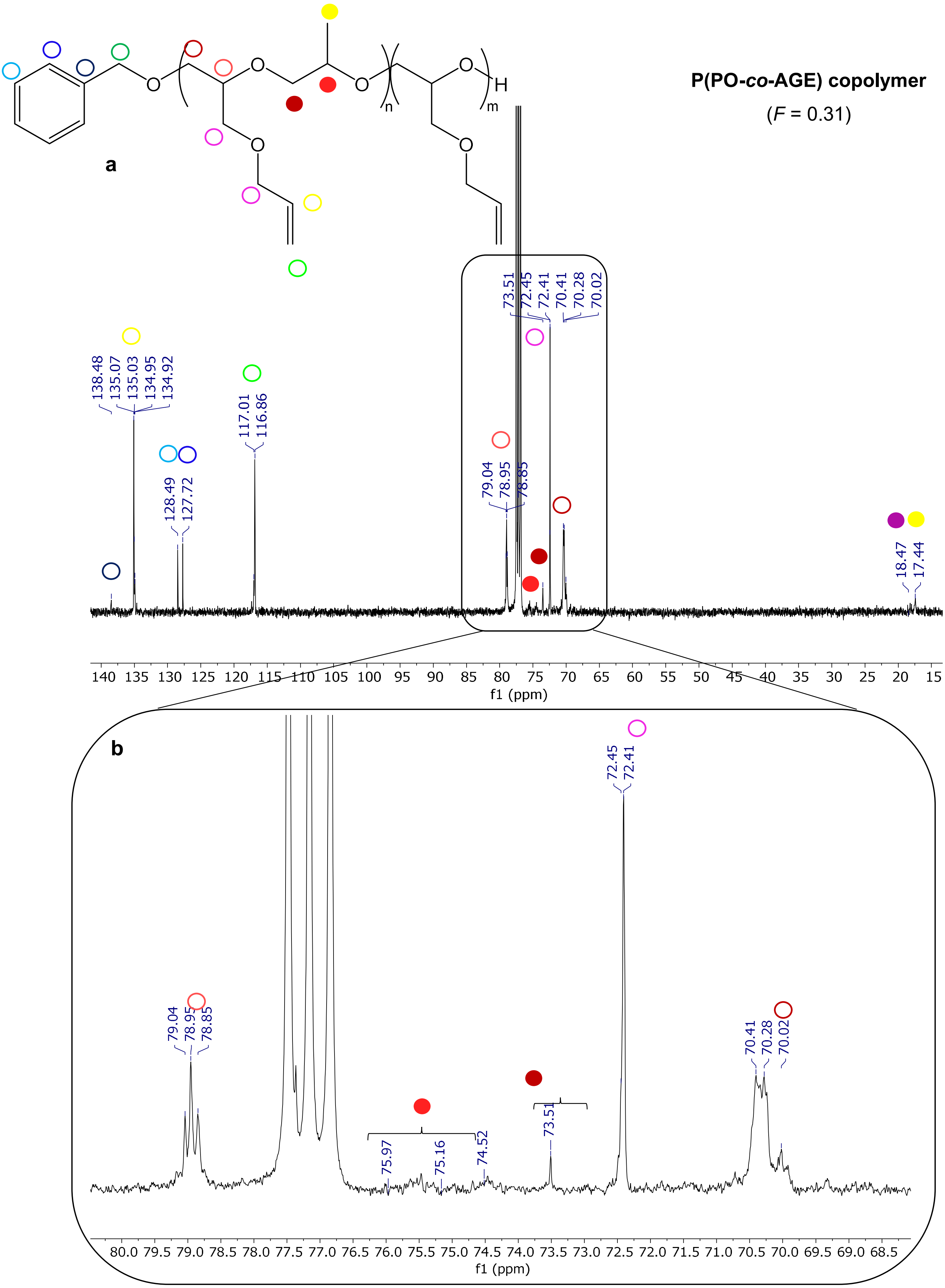

Supplementary Figure 14. (**a**) ^{13}C NMR spectrum ($CDCl_3$, 400 MHz) of P(PO-*co*-AGE) crude media (**b**) with a focus on the methine and methylene regions of P(PO-*co*-AGE) copolymer.

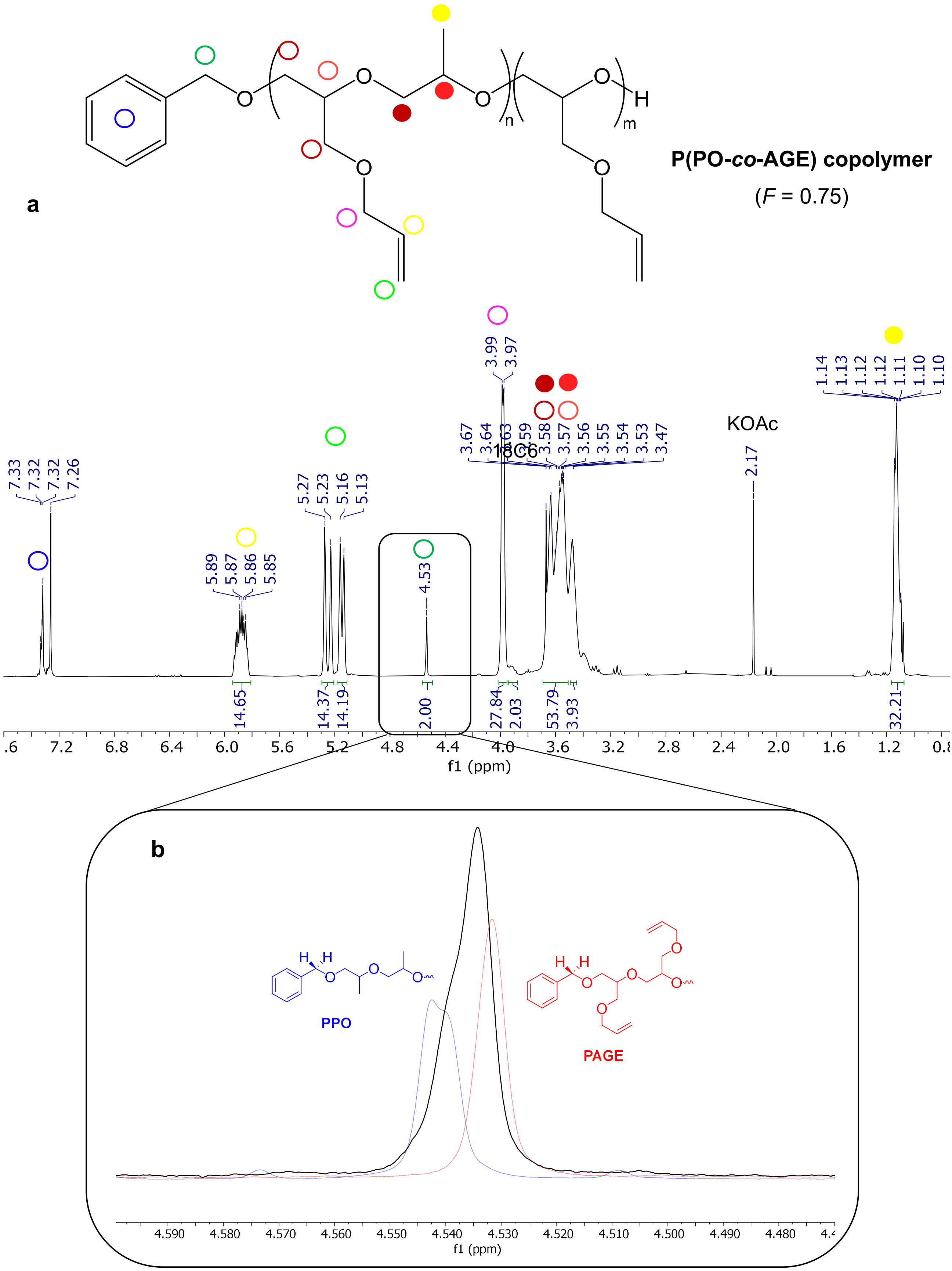

Supplementary Figure 15. (**a**) ^{1}H NMR spectrum ($CDCl_3$, 400 MHz) of P(PO-*co*-AGE) crude media. Conditions: $[PO+AGE]_0/[BnOH]_0$ = 25 with *F* of 0.75 after full conversion. (**b**) overlay ^{1}H NMR spectra to compare the α-phenyloxy end-groups of P(PO-*co*-AGE) (black spectrum) with the end-groups of PAGE (red spectrum) and PPO (blue spectrum).

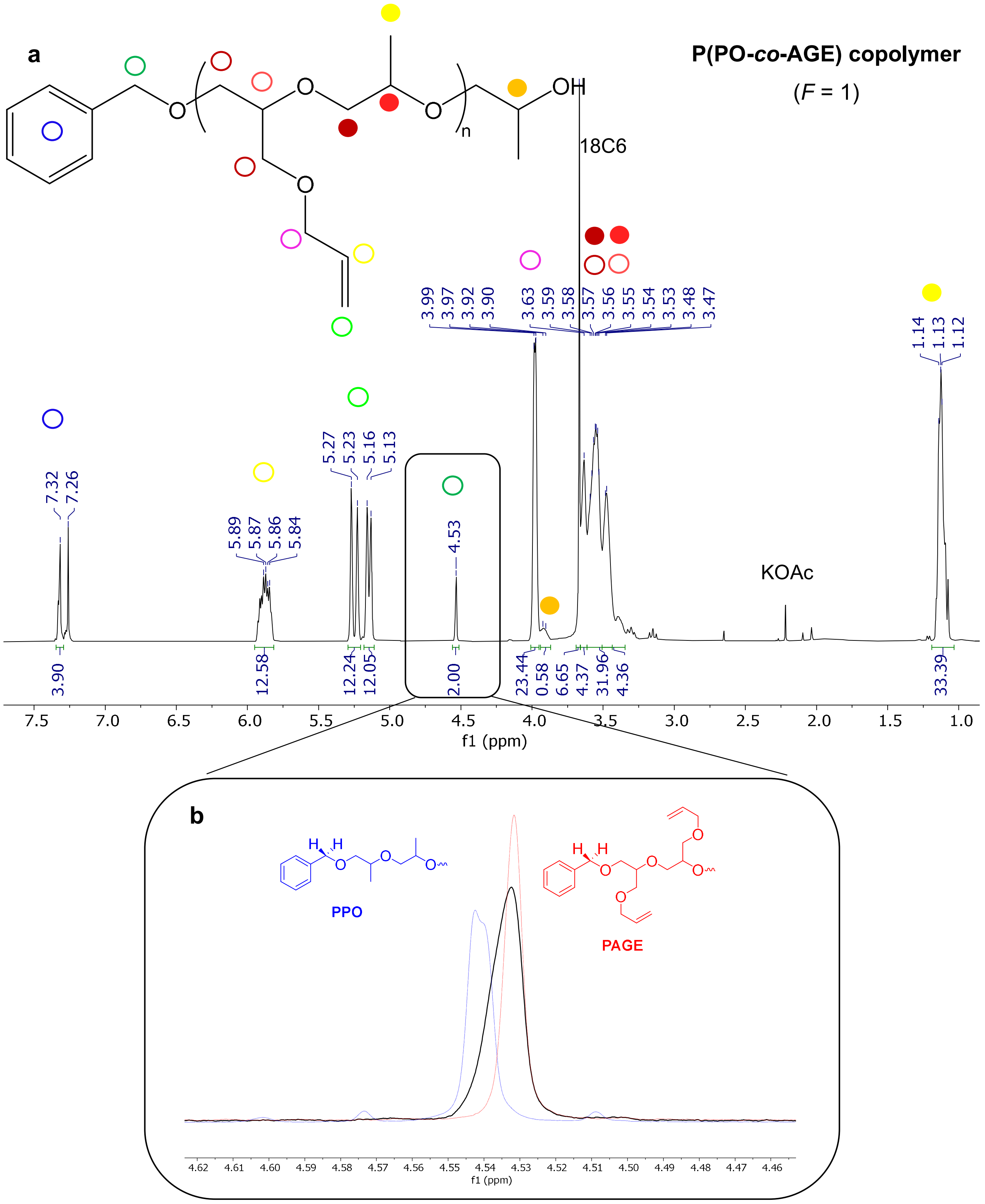

Supplementary Figure 16. (**a**) ^{1}H NMR spectrum ($CDCl_3$, 400 MHz) of P(PO-*co*-AGE) crude media. Conditions: $[PO+AGE]_0/[BnOH]_0$ = 25 with *F* of 1 after full conversion. (**b**) overlay ^{1}H NMR spectra to compare the α-phenyloxy end-groups of P(PO-*co*-AGE) (black spectrum) with the end-groups of PAGE (red spectrum) and PPO (blue spectrum).

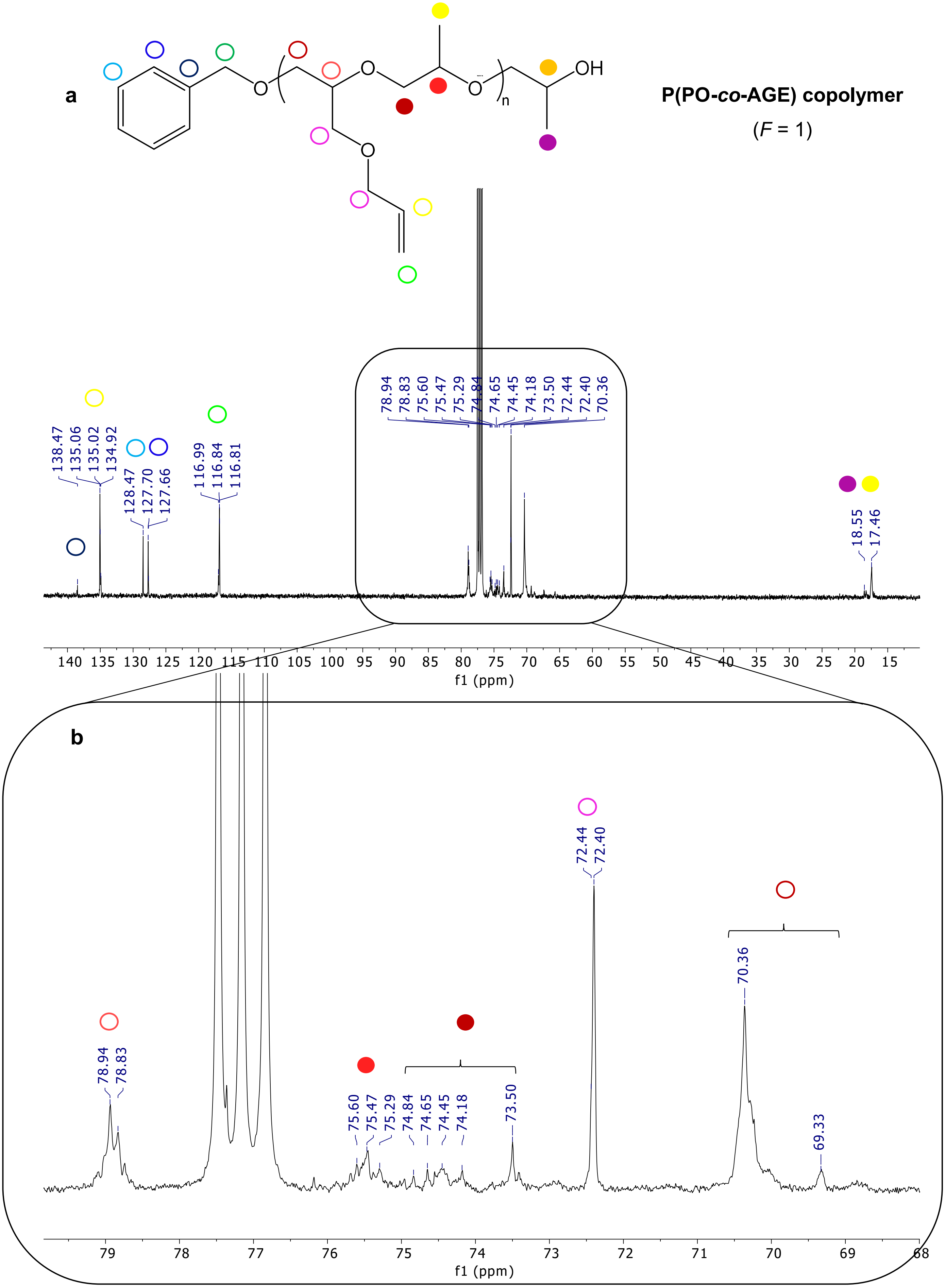

Supplementary Figure 17. (**a**) ^{13}C NMR spectrum ($CDCl_3$, 400 MHz) of P(PO-*co*-AGE) crude media (**b**) with a focus on the methine and methylene regions of P(PO-*co*-AGE) copolymer.

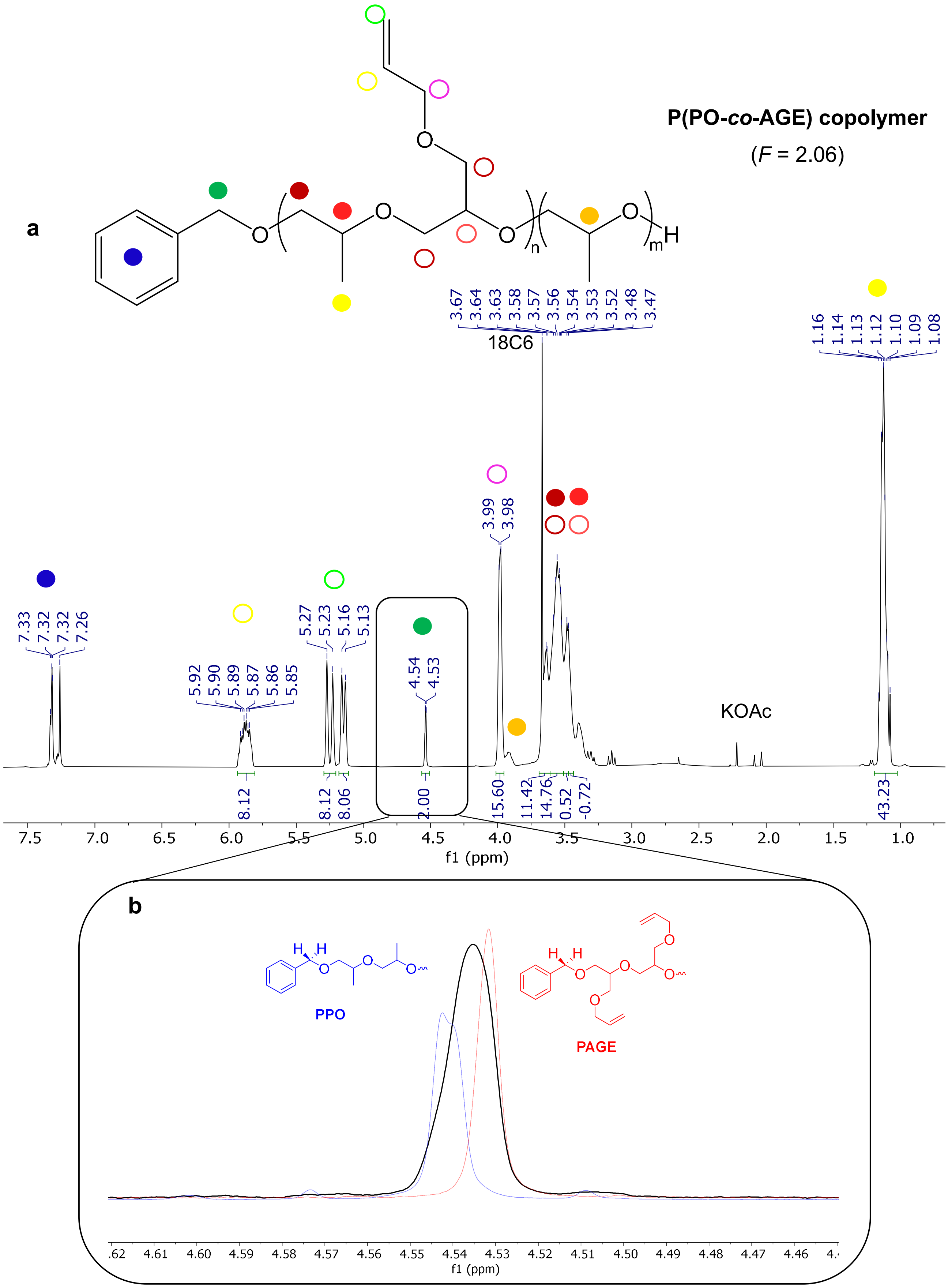

Supplementary Figure 18. (**a**) ^{1}H NMR spectrum ($CDCl_3$, 400 MHz) of P(PO-*co*-AGE) crude media. Conditions: $[PO+AGE]_0/[BnOH]_0$ = 25 with *F* of 2.06 after full conversion. (**b**) overlay ^{1}H NMR spectra to compare the α-phenyloxy end-groups of P(PO-*co*-AGE) (black spectrum) with the end-groups of PAGE (red spectrum) and PPO (blue spectrum).

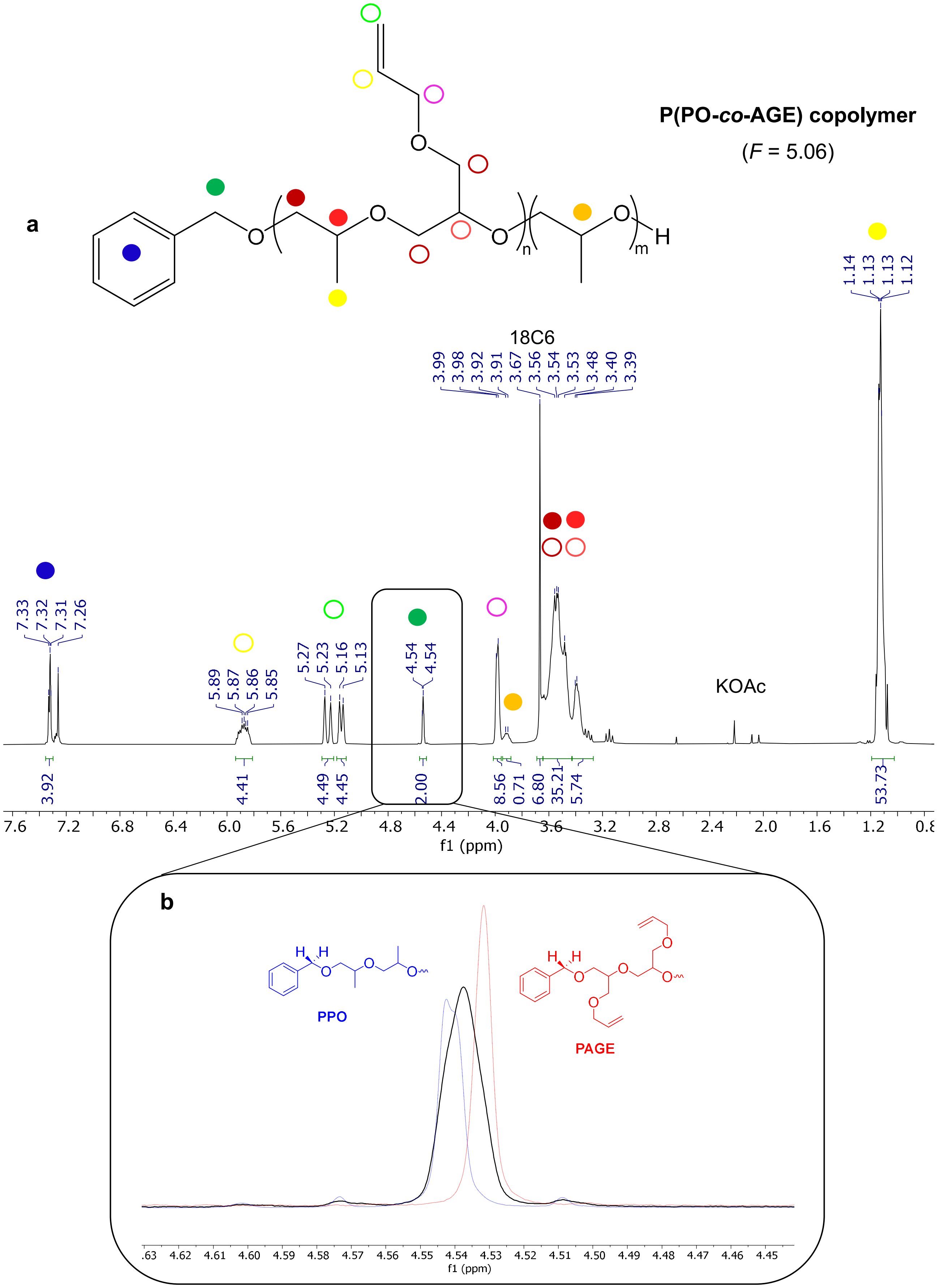

Supplementary Figure 19. (**a**) ^{1}H NMR spectrum ($CDCl_3$, 400 MHz) of P(PO-*co*-AGE) crude media. Conditions: $[PO+AGE]_0/[BnOH]_0$ = 25 with *F* of 5.06 after full conversion. (**b**) overlay ^{1}H NMR spectra to compare the α-phenyloxy end-groups of P(PO-*co*-AGE) (black spectrum) with the end-groups of PAGE (red spectrum) and PPO (blue spectrum).

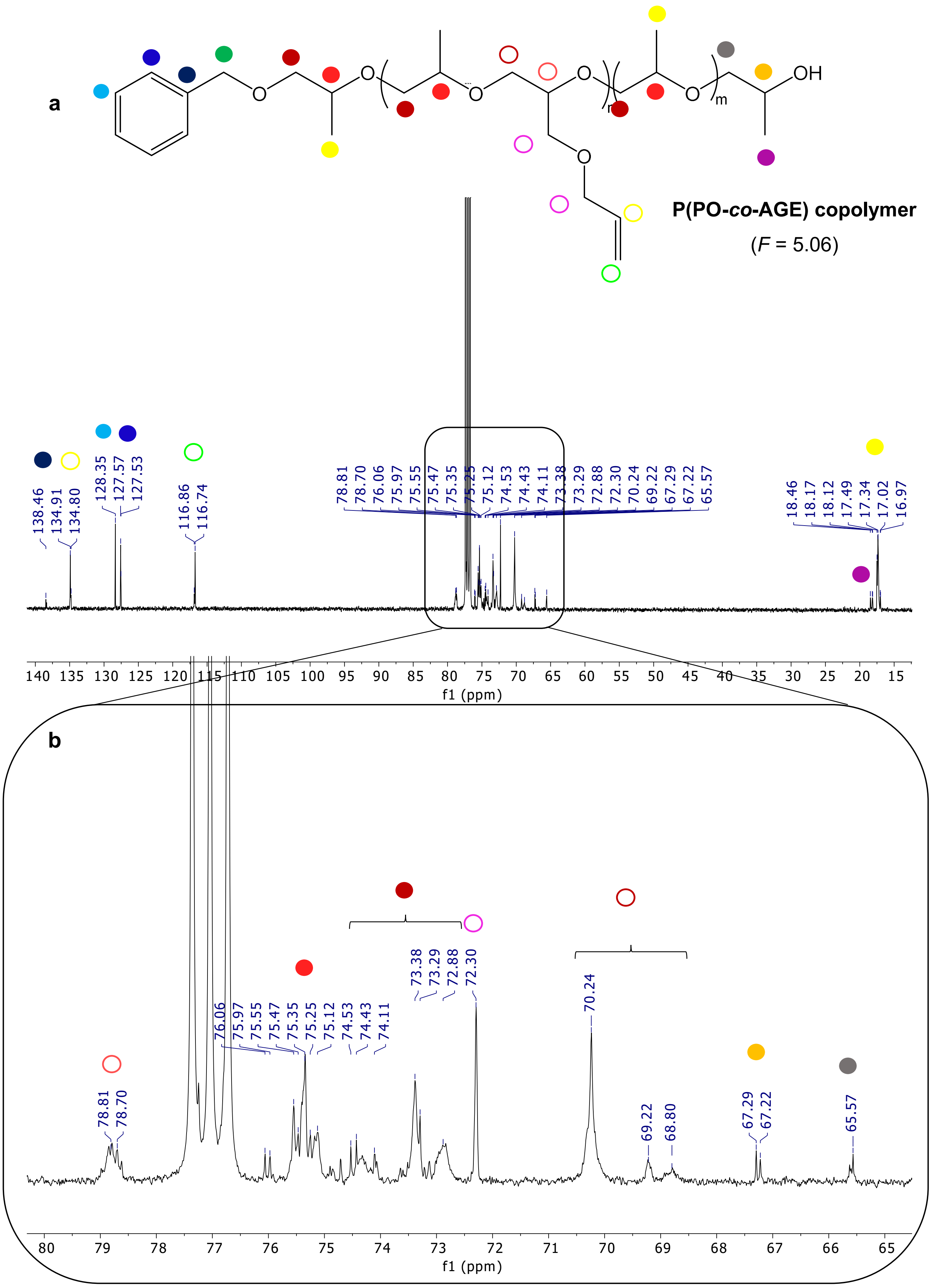

Supplementary Figure 20. (**a**) ^{13}C NMR spectrum ($CDCl_3$, 400 MHz) of P(PO-*co*-AGE) crude media (**b**) with a focus on the methine and methylene regions of P(PO-*co*-AGE) copolymer.

2.10. MALDI-ToF MS spectrum of PPO-*b*-PAGE Diblock Copolymer.

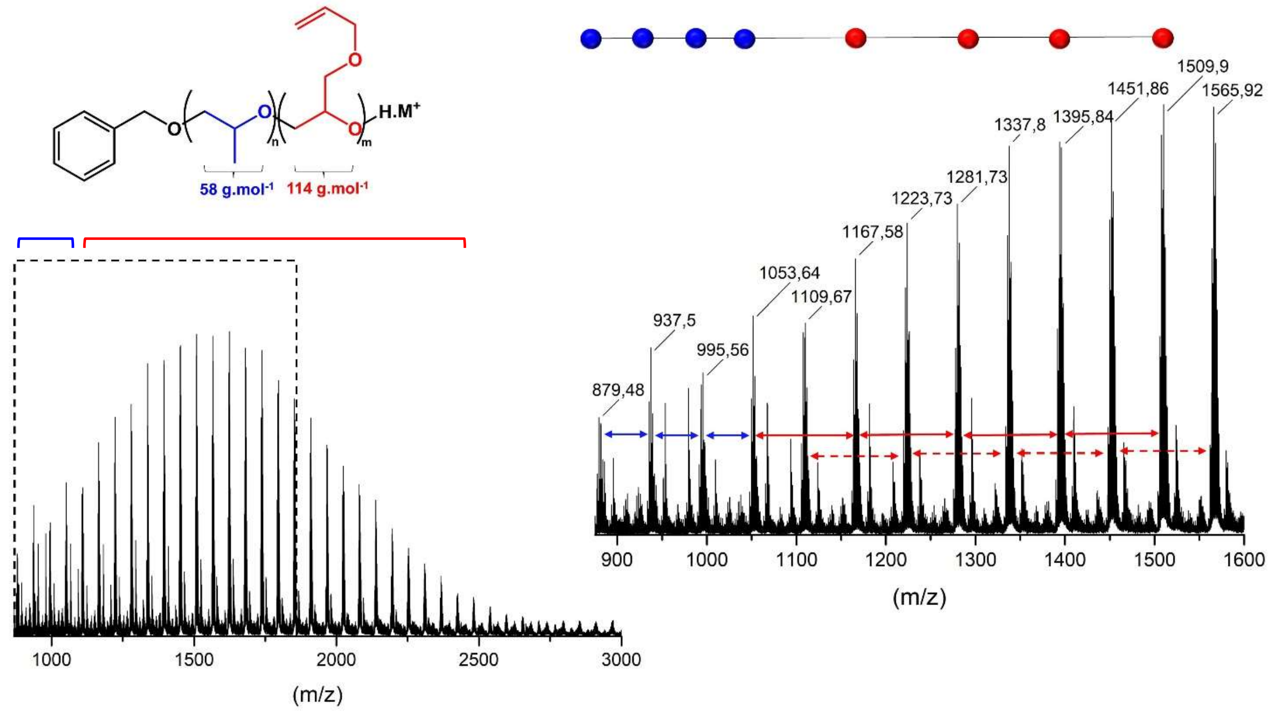

Supplementary Figure 21. Full MALDI-ToF MS spectrum of PPO-*b*-PAGE diblock copolymer crude media after full conversion. Conditions: $[PO+AGE]_0/[BnOH]_0$ = 25 with *F* of 1. Polymer sample was dissolved in THF to obtain 1 $mg.mL^{-1}$ solution. The insets show the structure of the PPO-*b*-PAGE diblock copolymer (top left) and expanded view of the *m/z* range 875–1600, in which oligomers are observed (top right). All peaks correspond to $C_7H_8OH(C_3H_6O)_n(C_6H_{10}O_2)_m.Na+$.

2.11. SEC traces, ^{1}H and ^{13}C NMR spectra of PPO-*b*-PAGE Diblock Copolymer.

a

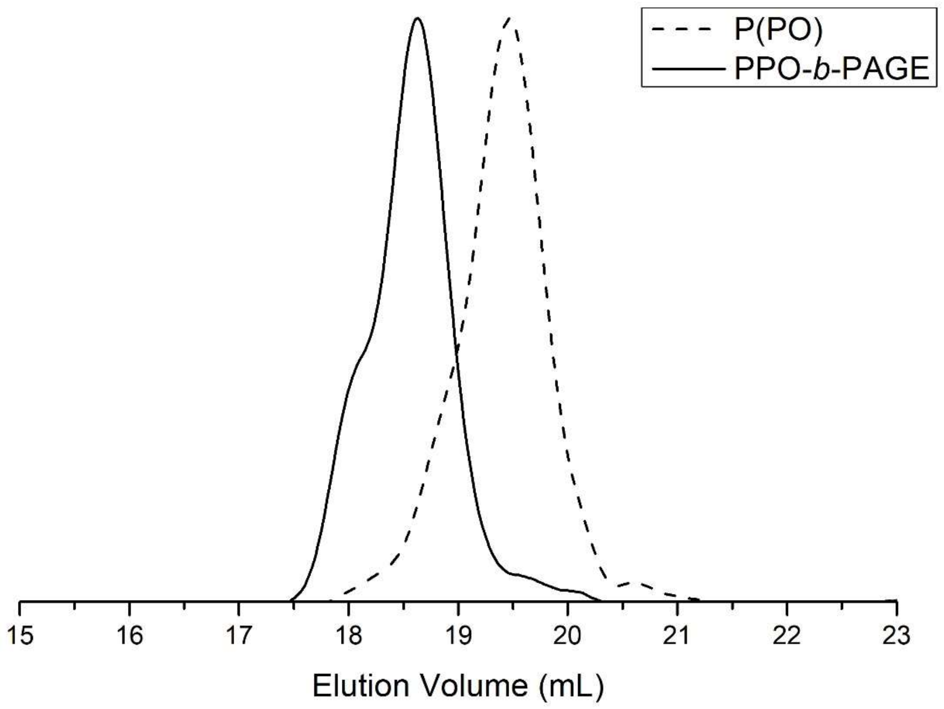

b

Feed	$\overline{DP}_{PO}$[a]	$\overline{DP}_{AGE}$[a]	M_n (g.mol^{-1}) [b]	$Đ_M$[b]
1	13	0	860	1.30
2	0	13	2210	1.27

Supplementary Figure 22. (**a**) Evolution SEC traces of P(PO) oligomer precursor (dash line) and corresponding PPO-*b*-PAGE diblock copolymer (black line) crude media. (**b**) Table reports the synthesis of PPO-*b*-PAGE diblock copolymer by targeting a DP of ~25 with *F* = 1. [a] The degree of polymerization ($\overline{DP}$), after each feed was consumed, was confirmed by ^{1}H NMR spectroscopy using the ratio between the integration of the methylene protons of the α-phenyloxy copolymers end-group and the allyl or methyl protons of the two different incorporated monomers (15 mg of polymer sample in 0.6 mL of $CDCl_3$). [b] determined by SEC in THF (15 mg/mL) at 40 °C relative to polystyrene (PS) standards.

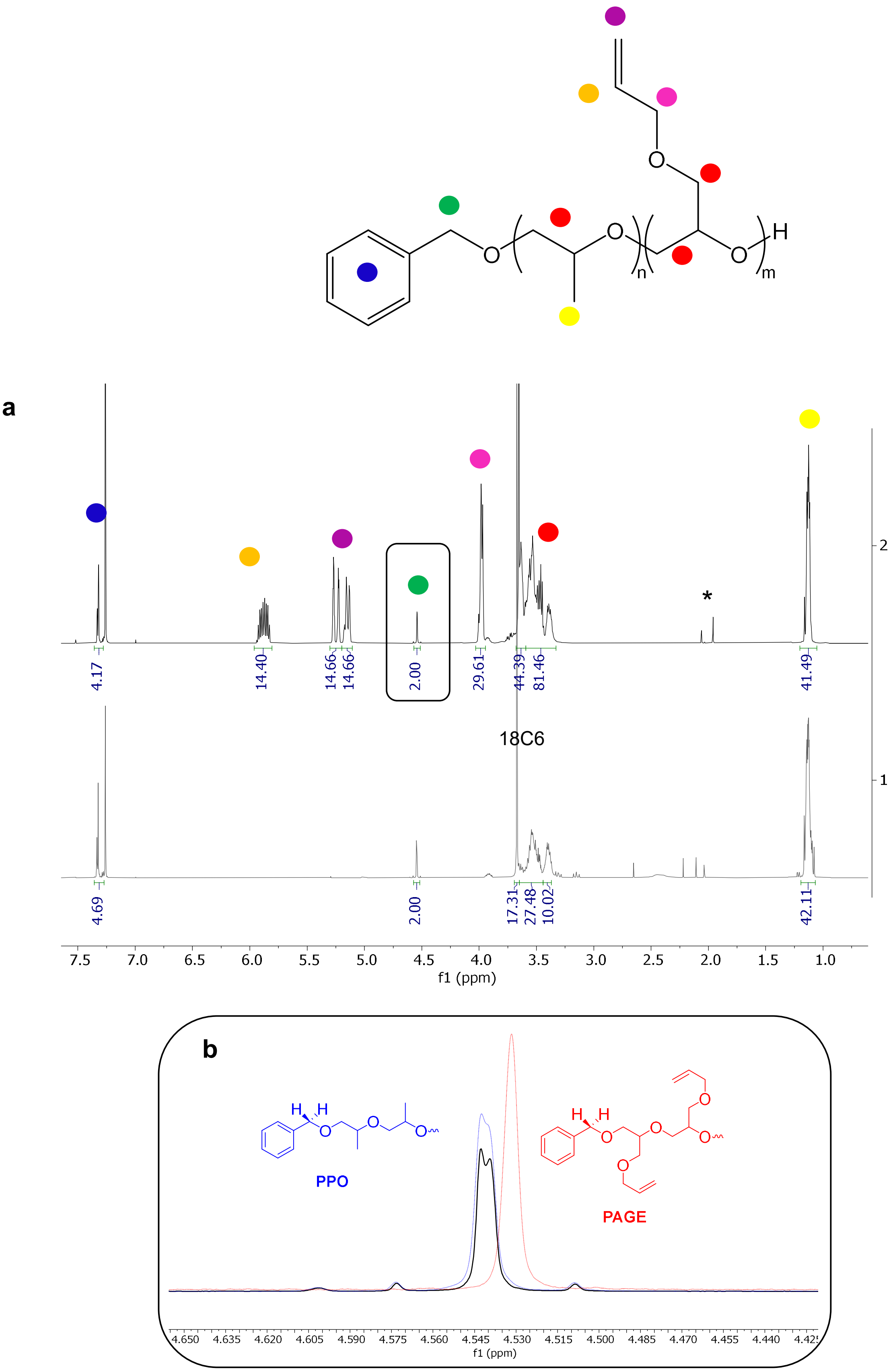

Supplementary Figure 23. (**a**) Stacked ^{1}H NMR spectra ($CDCl_3$, 400 MHz) of P(PO) oligomer precursor (bottom) and PPO-*b*-PAGE diblock copolymer crude media. Conditions: $[PO+AGE]_0/[BnOH]_0 = 25$ with F of 1 after full conversion. (*) Signals associated with the presence of impurities in crude products. (**b**) overlay ^{1}H NMR spectra to compare the α-phenyloxy end-groups of PPO-*b*-PAGE diblock copolymer (black spectrum) with the end-groups of PAGE (red spectrum) and PPO (blue spectrum).

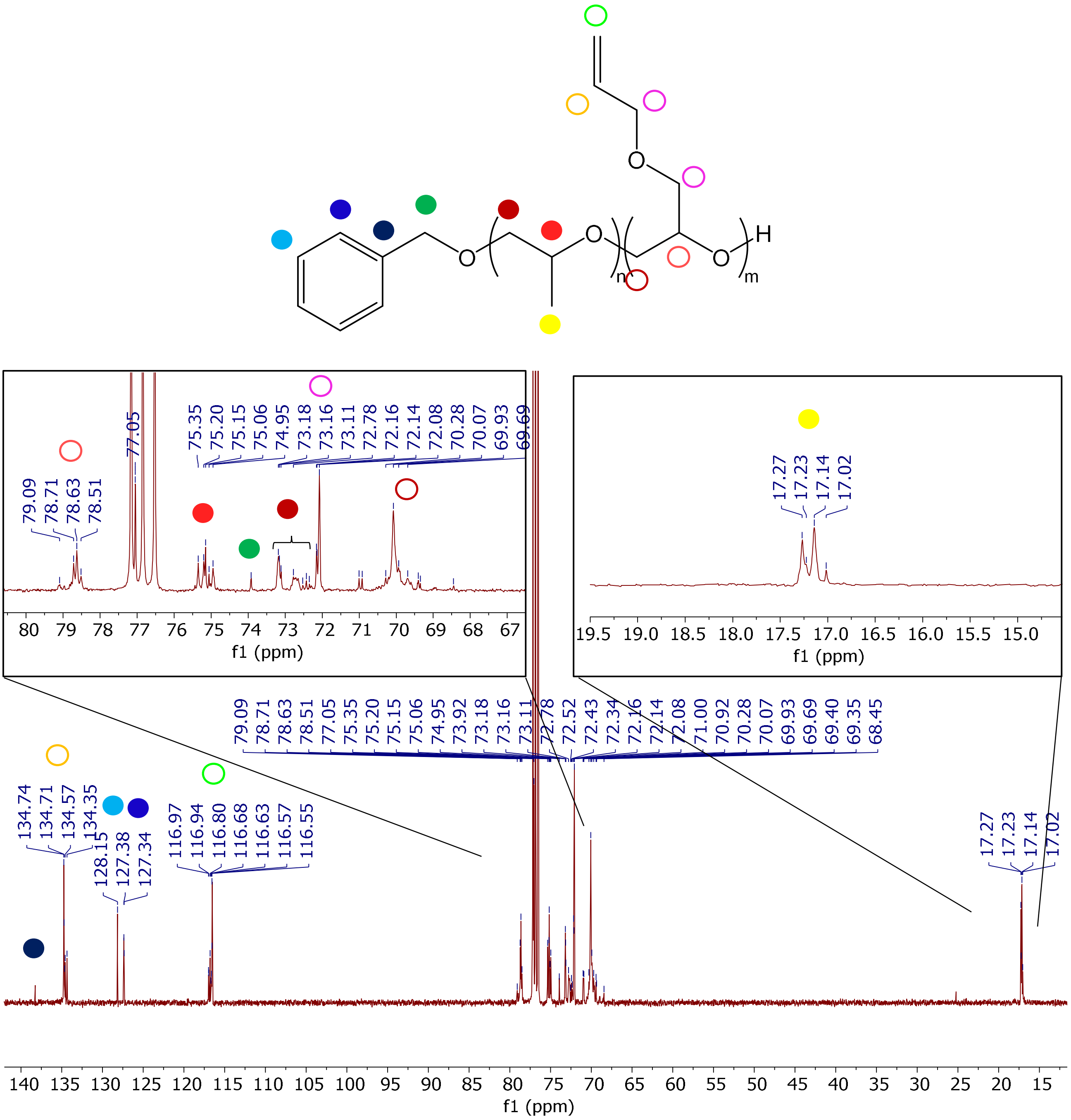

Supplementary Figure 24. ^{13}C NMR spectrum ($CDCl_3$, 400 MHz) of PPO-*b*-PAGE crude media with a focus on the methyl, methine and methylene carbon regions of PPO-*b*-PAGE diblock copolymer.

2.12. ^{1}H and ^{13}C NMR spectra of gradient P(PO-*co*-AGE) copolymer.

a

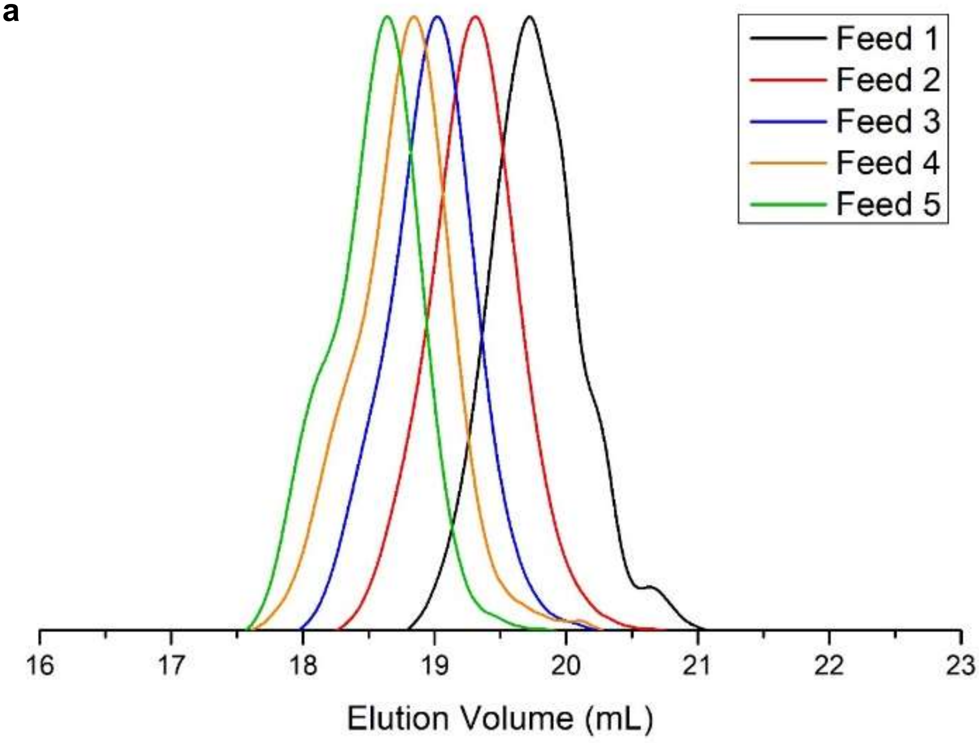

b

Feed	$\overline{DP}_{PO}$ [a]	$\overline{DP}_{AGE}$ [a]	M_n (g.mol^{-1}) [b]	$Đ_M$ [b]
1	0	5	515	1.25
2	1	4	920	1.26
3	4	2	1360	1.26
4	5	1	1760	1.27
5	3.5	0.5	2200	1.25

Supplementary Figure 25. (**a**) SEC chromatograms of gradient P(PO-*co*-AGE) copolymer crude media recorded at different stages of PO and AGE copolymerization. (**b**) Table with details on synthesis of gradient P(PO-*co*-AGE) copolymer. The synthesis was carried out with successive feed additions of monomers. Each feed addition was carried out after monitoring by NMR full conversion of all monomers, which occurred after 48-72 h. The total quantities of monomers (vs. initiator) were calculated to target a final DP ~25 and *F* ($[PO]_0/[AGE]_0$) = 1. [a] The degree of polymerization ($\overline{DP}$), after each feed was consumed, was confirmed by ^{1}H NMR spectroscopy using the ratio between the integration of the methylene protons of the α-phenyloxy copolymers end-group and the allyl or methyl protons of the two different incorporated monomers (10-13 mg of polymer sample in 0.6 mL of $CDCl_3$). [b] Determined by SEC in THF (10-13 mg/mL) at 40 °C relative to polystyrene (PS) standards.

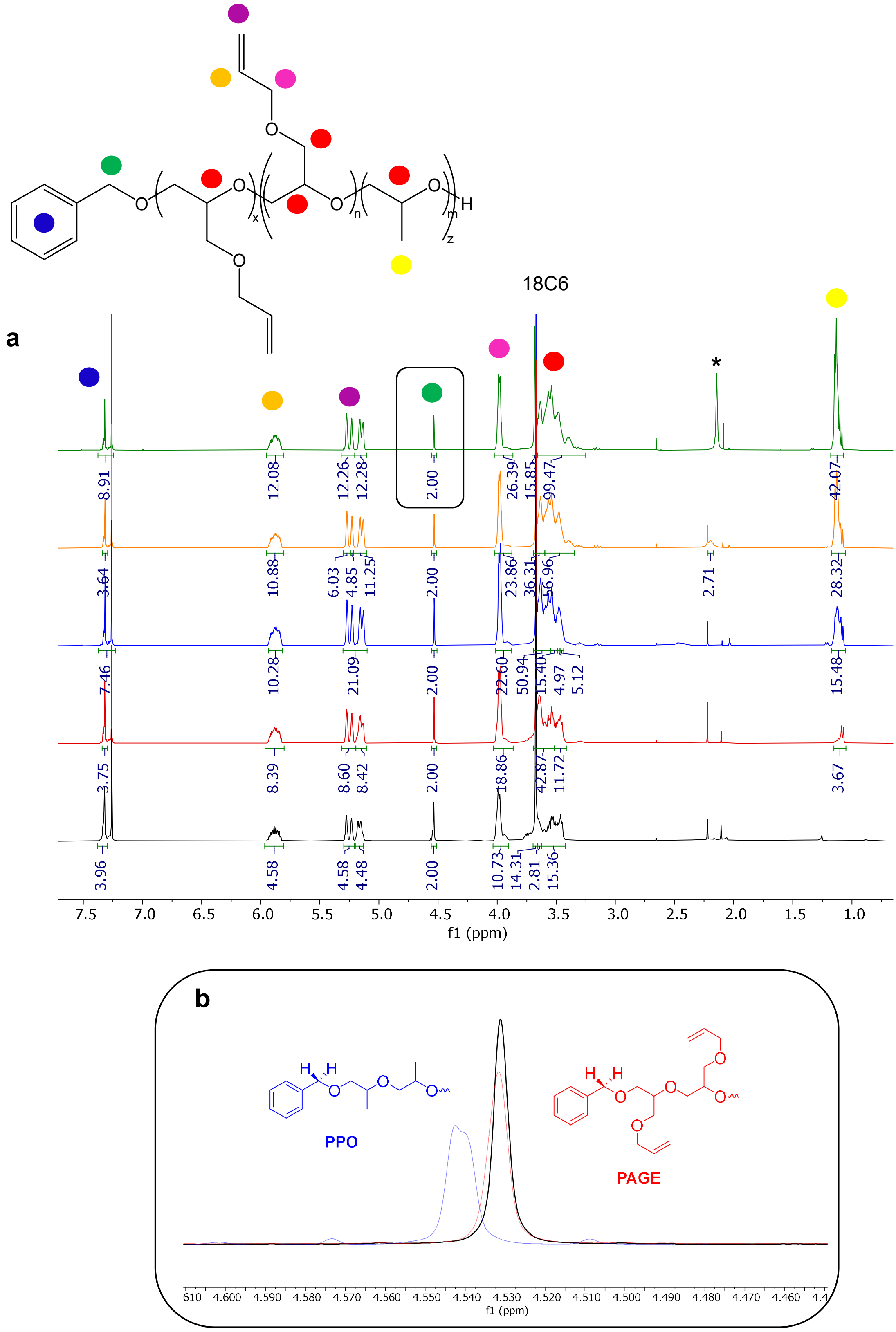

Supplementary Figure 26. (**a**) Stacked ^{1}H NMR spectra ($CDCl_3$, 400 MHz) of gradient P(PO-*co*-AGE) copolymer crude media during the chain growth process. (*) Signals associated with the presence of impurities in crude products. (**b**) overlay ^{1}H NMR spectra to compare the α-phenyloxy end-groups of gradient P(PO-*co*-AGE) copolymer (black spectrum) with the end-groups of PAGE (red spectrum) and PPO (blue spectrum).

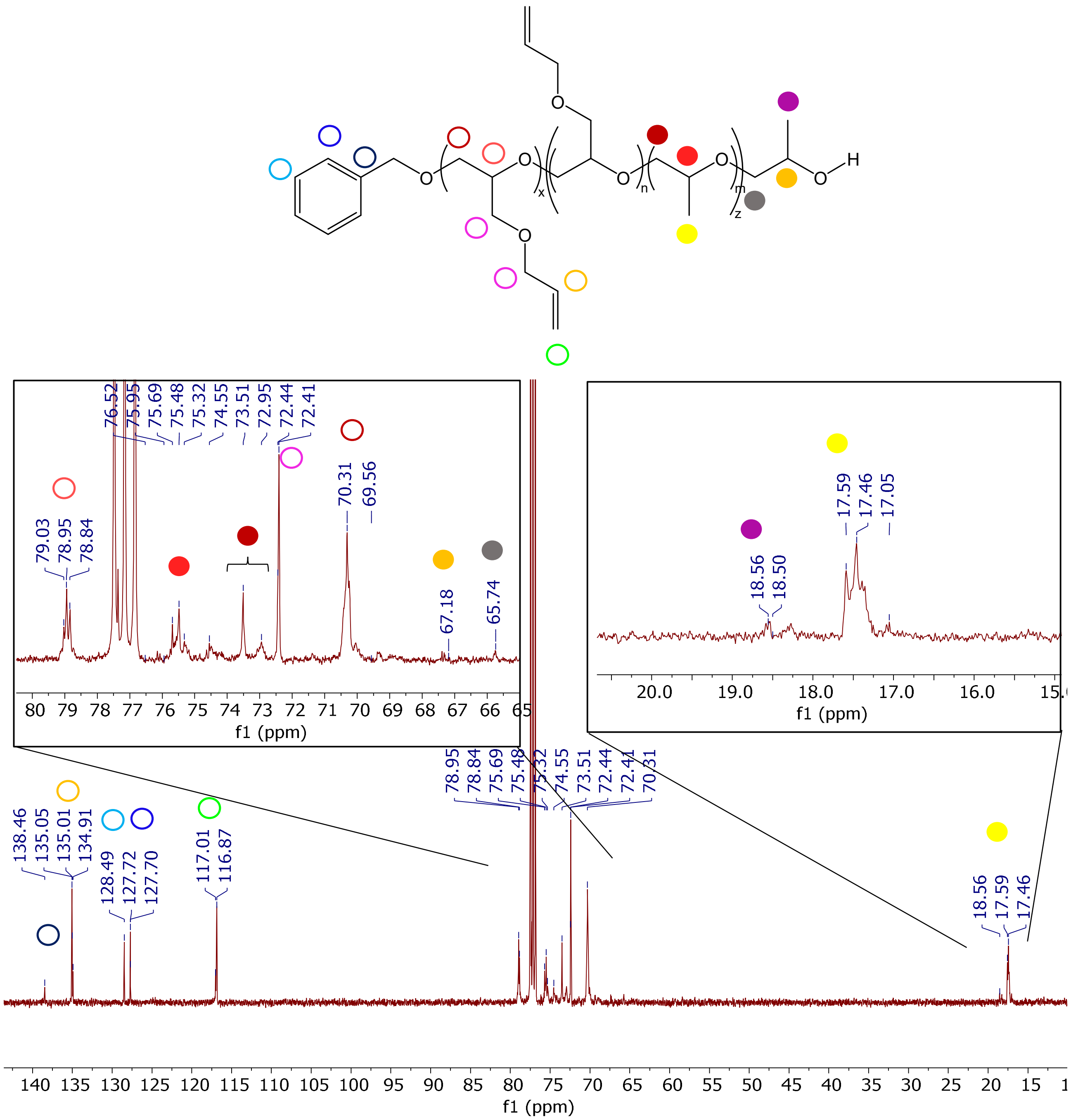

Supplementary Figure 27. ^{13}C NMR spectrum ($CDCl_3$, 400 MHz) of gradient P(PO-*co*-AGE) crude media with a focus on the methyl, methine and methylene carbon regions of gradient P(PO-*co*-AGE) copolymer.

2.13. Comparison of ^{13}C NMR spectra of PO/AGE Copolymers.

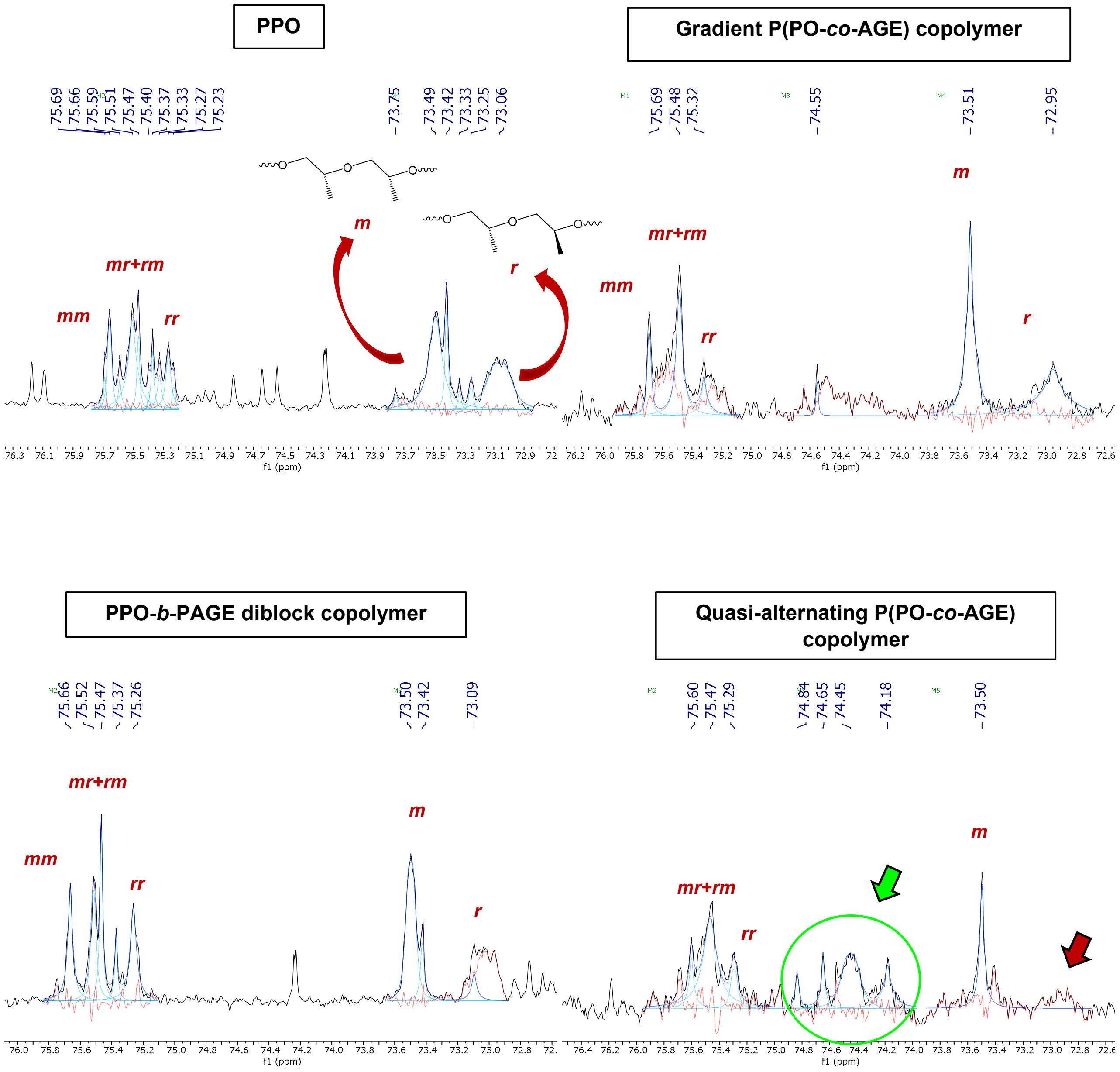

Supplementary Figure 28. Comparison of ^{13}C NMR spectra ($CDCl_3$, 101 MHz) of the crude PPO and the corresponding diblock, gradient and quasi-alternating PO/AGE copolymers. Zoom area showing the regions that correspond to the backbone methine carbon. The cross-peak region associated with mixed heterotriad sequences is tagged in green. The *m* and *r* refer to the meso and racemic.

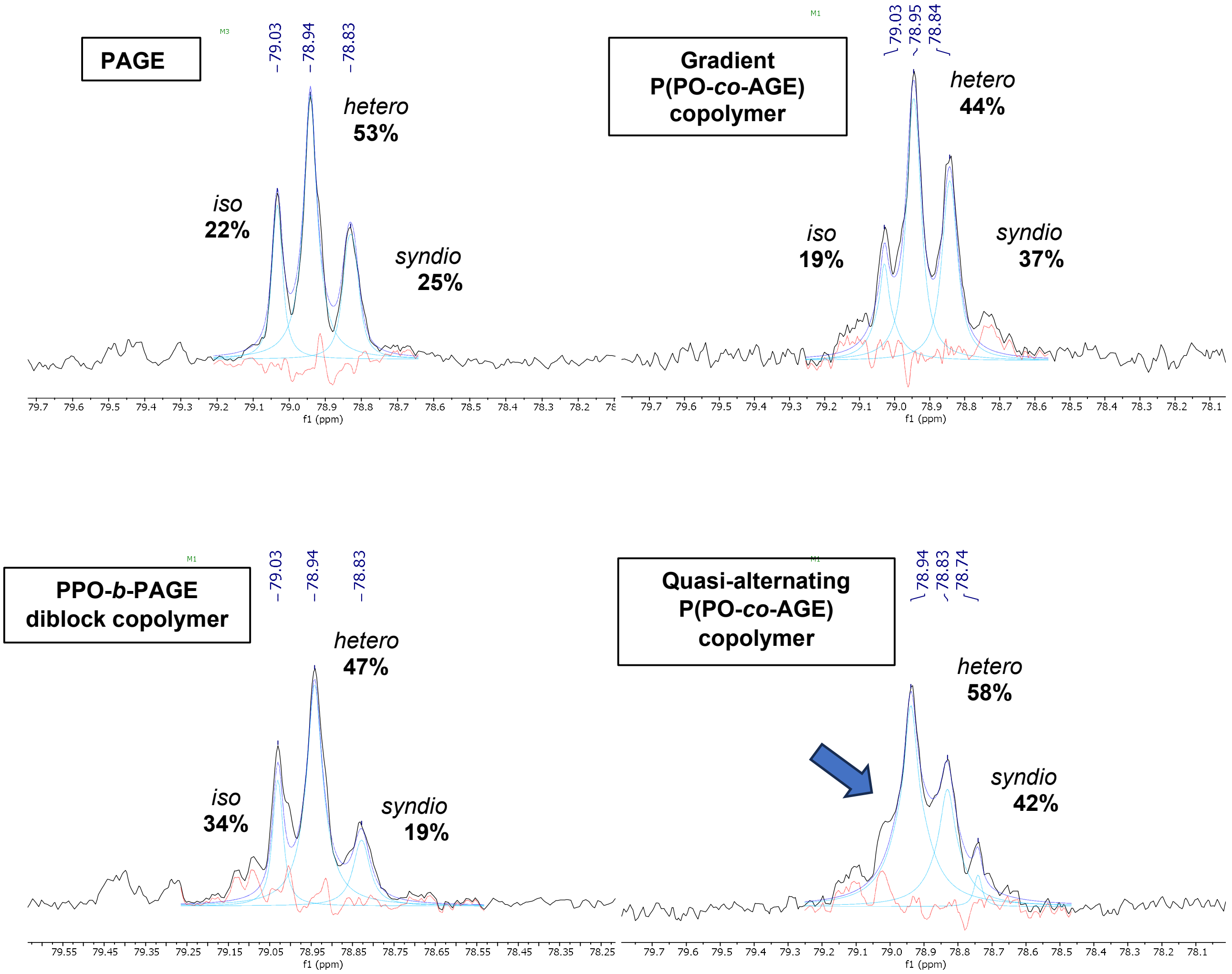

Supplementary Figure 29. Zoom area showing the regions that correspond to the backbone methine carbon on the AGE repeat units.

3. Supplementary Equations

3.1. Fineman-Ross Copolymerization Equation.

$$y = r_1 \cdot x + r_2$$

$$y = \frac{f_1(1 - 2 \cdot F_1)}{(1 - f_1) \cdot F_1} \qquad x = \frac{{f_1}^2(F_1 - 1)}{(1 - f_1)^2 \cdot F_1}$$

$$F_1 = \frac{d(m_1)}{d(m_1) + d(m_2)} \qquad f_1 = \frac{[M_1]}{[M_1] + [M_2]}$$

Supplementary Equation (1). Fineman-Ross copolymer composition equation used to extrapolate the reactivity ratios between comonomers. For this purpose, the copolymerization of the comonomers with different compositions (*F* ($[PO]_0/[AGE]_0$) of 0.1, 0.2, 0.31, 0.75, 2.06 and 5.06) were performed and the monomer composition in the obtained oligomers was examined at low conversion.

4. Supplementary References

1. Akkermans, R. L. C., Spenley, N. A. & Robertson, S. H. COMPASS III: automated fitting workflows and extension to ionic liquids. *Molecular Simulation*. **47**, 540–551 (2021).
2. Stephen L. Mayo, Barry D. Olafson, and William A. Goddard, J. Phys. Chem. 1990, 94, 26, 8897–8909
3. Fornaciari, C., Pasini, D. & Coulembier, O. Controlled Oxyanionic Polymerization of Propylene Oxide: Unlocking the Molecular-Weight Limitation by a Soft Nucleophilic Catalysis. *Macromol. Rapid Commun*. **43**, 2200424; 10.1002/marc.202200424 (2022).
4. Childers, M. I., Longo, J. M., Van Zee, N. J., LaPointe, A. M. & Coates, G. W. Stereoselective Epoxide Polymerization and Copolymerization. *Chem. Rev*. **114**, 8129−8152 (2014).
5. Rodriguez, C.G., Ferrier, R.C., Helenic, A. & Lynd, N.A. Ring-Opening Polymerization of Epoxides: Facile Pathway to Functional Polyethers via a Versatile Organoaluminum Initiator. *Macromolecules*. **50**, 3121–3130 (2017).
6. Fineman, M. & Ross, S. D. Linear method for determining monomer reactivity ratios in copolymerization. *J. Polym. Sci*. **5**, 259–262 (1959).
7. Pfeifer, S. & Lutz, J.-F. A facile procedure for controlling monomer sequence distribution in radical chain polymerizations. J*. Am. Chem. Soc*. **129**, 9542–9543 (2007).
8. Zamfir, M. & Lutz, J.-F. Ultra-precise insertion of functional monomers in chain-growth polymerizations. *Nat. Commun*. **3**, 1138 (2012).
