# Supplementary material for: Quasi-alternating copolymerization of oxiranes driven by a benign acetate-based catalyst
Source: Commun Chem. 2023 Oct 28;6:235. doi: 10.1038/s42004-023-01031-z (PMC10613202; doi:10.1038/s42004-023-01031-z)
Supplement: Supplementary file 2 — Description of Additional Supplementary Files [file 42004_2023_1031_MOESM2_ESM.pdf]

Description of Additional Supplementary Files

File name: Supplementary Data 1

Description: ^{1}H and ^{13}C NMR spectra data for homopolymers, all copolymers and monomer mixtures.
