# Supplementary material for: Quasi-alternating copolymerization of oxiranes driven by a benign acetate-based catalyst
Source: Commun Chem. 2023 Oct 28;6:235. doi: 10.1038/s42004-023-01031-z (PMC10613202; doi:10.1038/s42004-023-01031-z)
Supplement: Supplementary file 3 — Supplementary Data 1 [file 42004_2023_1031_MOESM3_ESM.docx]

**Supplementary Information (Supplementary Data 1)**

**Quasi-Alternating Copolymerization of Oxiranes Driven by a Benign Acetate-based Catalyst**

Charlotte Fornaciari^1,2^, Vincent Lemaur^3^, Dario Pasini^2^*, Olivier Coulembier^1^*

^1^ Laboratory of Polymeric and Composite Materials (LPCM), Center of Innovation and Research in Materials and Polymers (CIRMAP), University of Mons, Place du Parc, 20, Mons 7000, Belgium.

^2^ Department of Chemistry, University of Pavia, Viale Taramelli, 10, Pavia 27100, Italy.

^3^ Laboratory for Chemistry of Novel Materials, Center for Innovation and Research in Materials and Polymers (CIRMAP), University of Mons, Place du Parc, 20, 7000 Mons, Belgium.

E-mail: [olivier.coulembier@umons.ac.be](mailto:olivier.coulembier@umons.ac.be)

[dario.pasini@unipv.it](mailto:dario.pasini@unipv.it)

^1^H NMR spectrum (CDCl_3_, 500 MHz) of the crude product between BnOH and PO.

^1^H NMR spectrum (CDCl_3_, 400 MHz) of the crude PPO after full conversion.


^1^H NMR kinetics (CDCl_3_, 500 MHz) of the PO polymerization during the early stage of the reaction.

^13^C NMR spectrum (CDCl_3_, 101 MHz) of the crude PPO.

^1^H NMR spectrum (CDCl_3_, 400 MHz) of the crude PAGE after full conversion.

^1^H NMR kinetic studies (CDCl_3_, 500 MHz) of the AGE polymerization during the early stage of the reaction.

^13^C NMR spectrum (CDCl_3_, 101 MHz) of the crude PAGE.

^1^H NMR spectrum (CDCl_3_, 400 MHz) of P(PO-*co*-AGE) crude media with *F* = 0.1

Full stacked ^1^H NMR spectra (CDCl_3_, 500 MHz) of P(PO-*co*-AGE) crude media with *F* = 0.1 in function of the time.

^13^C NMR spectrum (CDCl_3_, 101 MHz) of P(PO-*co*-AGE) crude media with *F* = 0.1.


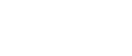


^1^H NMR spectrum (CDCl_3_, 400 MHz) of P(PO-*co*-AGE) crude media with *F* of 0.2.

^1^H NMR kinetic studies (CDCl_3_, 500 MHz) of the PO and AGE copolymerization with *F* = 0.2 in the early stage of copolymerization.

^13^C NMR spectrum (CDCl_3_, 101 MHz) of P(PO-*co*-AGE) crude media with *F* = 0.2.

^1^H NMR spectrum (CDCl_3_, 400 MHz) of P(PO-*co*-AGE) crude media with *F* of 0.31.

^1^H NMR kinetic studies (CDCl_3_, 500 MHz) of the copolymerization of PO and AGE with *F* = 0.31 in the early stage of copolymerization.

^13^C NMR spectrum (CDCl_3_, 101 MHz) of P(PO-*co*-AGE) crude media with *F* = 0.31.

^1^H NMR spectrum (CDCl_3_, 400 MHz) of P(PO-*co*-AGE) crude media with *F* of 0.75.

^1^H NMR kinetic studies (CDCl_3_, 500 MHz) of the copolymerization of PO and AGE with *F* = 0.75 in the early stage of copolymerization.

^13^C NMR spectrum (CDCl_3_, 101 MHz) of P(PO-*co*-AGE) crude media with *F* = 0.75.

^1^H NMR spectrum (CDCl_3_, 400 MHz) of P(PO-*co*-AGE) crude media with *F* of 1.

^1^H NMR kinetic studies (CDCl_3_, 500 MHz) of the copolymerization of PO and AGE with *F* = 1 in the early stage of copolymerization.


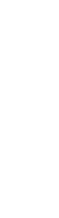

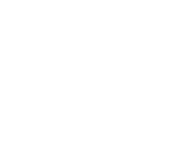
 ^13^C NMR spectrum (CDCl_3_, 101 MHz) of P(PO-*co*-AGE) crude media with *F* = 1.

^1^H NMR spectrum (CDCl_3_, 400 MHz) of P(PO-*co*-AGE) crude media with *F* of 2.06.

^1^H NMR kinetic studies (CDCl_3_, 500 MHz) of the copolymerization of PO and AGE with *F* = 2.06 in function of the time.

^13^C NMR spectrum (CDCl_3_, 101 MHz) of P(PO-*co*-AGE) crude media with *F* = 2.06

^1^H NMR spectrum (CDCl_3_, 400 MHz) of P(PO-*co*-AGE) crude media with *F* of 5.06.

^1^H NMR spectra overlay (CDCl_3_, 500 MHz) of P(PO-*co*-AGE) crude media with *F* = 5.06 in function of the time.


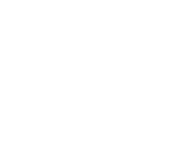


^13^C NMR spectrum (CDCl_3_, 101 MHz) of P(PO-*co*-AGE) crude media with *F* = 5.06.

Stacked ^1^H NMR spectra (CDCl_3_, 400 MHz) of of P(PO) oligomer precursor (bottom) PPO-*b*-PAGE block copolymer crude media.

^13^C NMR spectrum (CDCl_3_, 400 MHz) of PPO-*b*-PAGE crude media.

Stacked ^1^H NMR spectra (CDCl_3_, 400 MHz) of gradient P(PO-*co*-AGE) copolymer crude media during the chain growth process.

^13^C NMR spectrum (CDCl_3_, 400 MHz) of gradient P(PO-*co*-AGE) crude media.

^1^H NMR spectrum (THF-*d*_8_, 500 MHz) of PO. Total concentration of [PO] = 8.6 M.

^1^H NMR spectrum (THF-*d*_8_, 500 MHz) of AGE. Total concentration of [AGE] = 8.6 M.


^1^H NMR spectrum (THF-*d*_8_, 500 MHz) of PO and AGE mixture. The molar ratio of the two monomers [PO]_0_/[AGE]_0_ = 0.1. Total mixture concentration of [PO+AGE] = 8.6 M.

^1^H NMR spectrum (THF-*d*_8_, 500 MHz) of PO and AGE mixture. The molar ratio of the two monomers [PO]_0_/[AGE]_0_ = 0.3. Total mixture concentration of [PO+AGE] = 8.6 M.

^1^H NMR spectrum (THF-*d*_8_, 500 MHz) of PO and AGE mixture. The molar ratio of the two monomers [PO]_0_/[AGE]_0_ = 0.5. Total mixture concentration of [PO+AGE] = 8.6 M.

^1^H NMR spectrum (THF-*d*_8_, 500 MHz) of PO and AGE mixture. The molar ratio of the two monomers [PO]_0_/[AGE]_0_ = 1. Total mixture concentration of [PO+AGE] = 8.6 M.

^1^H NMR spectrum (THF-*d*_8_, 500 MHz) of PO and AGE mixture. The molar ratio of the two monomers [PO]_0_/[AGE]_0_ = 3. Total mixture concentration of [PO+AGE] = 8.6 M.

^1^H NMR spectrum (THF-*d*_8_, 500 MHz) of PO and AGE mixture. The molar ratio of the two monomers [PO]_0_/[AGE]_0_ = 6. Total mixture concentration of [PO+AGE] = 8.6 M.

^1^H NMR spectrum (THF-*d*_8_, 500 MHz) of PO and AGE mixture. The molar ratio of the two monomers [PO]_0_/[AGE]_0_ = 9. Total mixture concentration of [PO+AGE] = 8.6 M.
